# MRPL12 K163 acetylation inhibits ccRCC via driving mitochondrial metabolic reprogramming

**DOI:** 10.1038/s41419-025-07896-3

**Published:** 2025-08-26

**Authors:** Xingzhao Ji, Fuyuan Xue, Ying Wang, Dexuan Gao, Jian Sun, Tianyi Dong, Qian Mu, Quanlin Xu, Shengnan Sun, Yi Liu, Qiang Wan

**Affiliations:** 1 Shandong Provincial Key Medical and Health Laboratory of cell metabolism, Central Hospital Affiliated to Shandong First Medical University, Jinan, Shandong 250021 China; 2 Department of Pulmonary and Critical Care Medicine, Shandong Provincial Hospital Affiliated to Shandong First Medical University, Jinan, Shandong 250021 China; 3 Medical Science and Technology Innovation Center, Shandong First Medical University & Shandong Academy of Medical Sciences, Jinan, Shandong 250021 China; 4 Department of Urological Surgery, Shandong Provincial Hospital Affiliated to Shandong First Medical University, Jinan, Shandong China; 5 Department of Breast and Thyroid Surgery, Shandong Provincial Hospital Affiliated to Shandong First Medical University, Jinan, Shandong China

**Keywords:** Cancer, Cancer metabolism

## Abstract

Renal cell carcinoma (RCC) is a common urological tumor, with clear cell renal cell carcinoma (ccRCC) being the most prevalent subtype. Metabolic reprogramming plays a critical role in ccRCC progression, making it a promising target for therapeutic intervention, though effective treatments remain unavailable. Our previous studies have shown that mitochondrial ribosomal protein L12 (MRPL12) contributes to various metabolic diseases, including diabetic kidney disease and HCC, by regulating mitochondrial biosynthesis. In this study, we demonstrated that MRPL12 is acetylated at lysine 163 (K163) in ccRCC cells, a key modification that influences its regulatory effect on mitochondrial metabolism. Mechanistically, we clarified that acetylation at the K163 site enhances mitochondrial biosynthesis by promoting MRPL12’s binding to POLRMT, which subsequently increases mitochondrial metabolism and suppresses cellular glycolysis. Additionally, we found that MRPL12 K163 acetylation levels were significantly downregulated in ccRCC and that restoring this acetylation inhibited ccRCC progression in both in vitro and in vivo models. Furthermore, we demonstrated that the acetyltransferase TIP60 and the deacetylase SIRT5 bind to MRPL12 and regulate its acetylation. These findings highlight K163 acetylation as a critical site for MRPL12-mediated regulation of mitochondrial metabolism and reveal that this modification inhibits renal cancer development by promoting mitochondrial biosynthesis, reducing glycolysis, and driving metabolic reprogramming. This study suggests a potential therapeutic strategy for targeting MRPL12 acetylation in ccRCC.

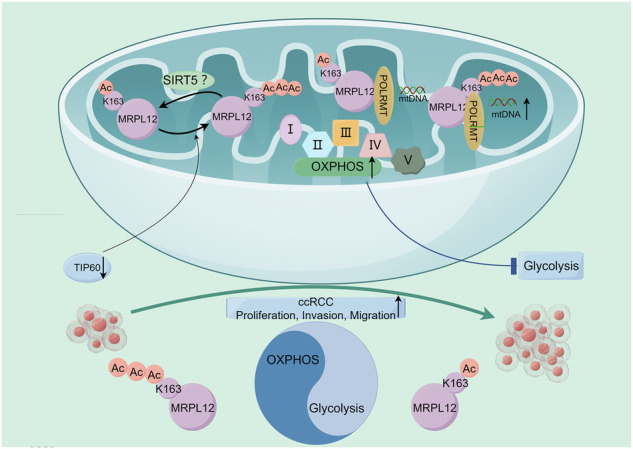

## Introduction

Renal cell carcinoma (RCC) is a common urological malignancy, ranking as the sixth most frequently diagnosed cancer in men and the tenth in women [1]. RCC is primarily classified into three subtypes: chromophobe RCC (chRCC), papillary RCC (pRCC), and the most common form, clear cell RCC (ccRCC), which accounts for approximately 75% of diagnosed cases [2]. Although ccRCC can often be detected early and treated effectively through surgical or ablative strategies, the prognosis remains suboptimal [3]. Current therapeutic options include tyrosine kinase inhibitors (TKIs), mTORC signaling inhibitors, and immune checkpoint inhibitors [4, 5]. However, despite initial efficacy, most patients eventually develop resistance to these treatments, leading to disease progression and, ultimately, mortality [6, 7]. This underscores the urgent need for more effective therapeutic targets to inhibit ccRCC progression.

Metabolic reprogramming is a hallmark of cancer progression, and targeting metabolic alterations presents a promising approach for cancer therapy [8]. Mitochondria play a crucial role in orchestrating these metabolic adaptations, promoting cellular growth and proliferation [9]. Clear cell RCC exhibits the hallmark features of the Warburg metabolism, characterized by significantly increased glycolysis and reduced oxidative phosphorylation capacity [10]. Metabolomic [11, 12], proteomic [11] and transcriptomic [12] studies have demonstrated elevated levels of glycolysis metabolites and enzymes. Notably, studies have shown that ectopic expression of glycolysis-antagonizing enzymes inhibits ccRCC tumor growth in xenograft models [13], underscoring the importance of metabolic reprogramming in ccRCC. However, effective targets to intervene in ccRCC glycolysis remain elusive.

Mitochondrial ribosomal proteins (MRPs) are essential for the structural and functional integrity of the mitochondrial ribosome [14]. As the first mitochondrial ribosomal protein discovered in mammalian cells, MRPL12 has recently been identified as a mitochondrial transcription factor, playing a crucial role in the regulation of mitochondrial biogenesis. It has been suggested that MRPL12 interacts with mitochondrial RNA polymerase to activate mtDNA transcription and regulate mitochondrial gene expression [15–17]. In our previous studies, we explored the role of MRPL12 in regulating mitochondrial metabolism across various metabolic diseases, including diabetic kidney disease, acute kidney injury, hepatocellular carcinoma, and lung adenocarcinoma. We found that MRPL12 drives mitochondrial metabolic reprogramming by modulating mitochondrial biogenesis, contributing to disease development and progression, making it a potential target for therapeutic intervention [18–24]. Our previous research concluded that MRPL12 plays a critical regulatory role in mitochondrial metabolism. However, it remains unclear whether MRPL12, as a key factor in mitochondrial biogenesis and metabolism, plays a critical role in ccRCC, which is characterized by a prominent Warburg effect.

Post-translational modifications (PTMs) are complex and fundamental regulators of biological processes [25]. Among PTMs, protein acetylation is a major mechanism that regulates protein function by influencing catalytic activity, protein-protein interactions, subcellular localization, and protein stability [26–30]. Mitochondrial protein acetylation has gained attention, with studies revealing that approximately 63% of mitochondrial proteins contain lysine acetylation sites [31]. Our previous research was the first to reveal that MRPL12 undergoes ubiquitination and phosphorylation, modifications directly linked to its role in mitochondrial metabolic regulation [20, 24]. However, it remains unreported whether MRPL12, as a mitochondrial protein, is subject to acetylation and whether this modification influences its biological function.

In this study, we revealed that MRPL12 undergoes acetylation at lysine 163 (K163), with significantly reduced levels of K163 acetylation observed in ccRCC tissues, which correlates strongly with poor prognosis. We further demonstrated that MRPL12 acetylation at K163 upregulates mitochondrial oxidative phosphorylation, inhibits glycolysis, and thereby affects the malignant phenotype of ccRCC cells in vivo and in vitro. Mechanistically, we found that K163 acetylation modulates MRPL12’s binding to mitochondrial RNA polymerase, influencing both mitochondrial biogenesis and glycolytic capacity in ccRCC cells. Finally, we identified key enzymes responsible for mediating the acetylation and deacetylation of MRPL12. Our findings suggest that MRPL12 K163 acetylation drives metabolic reprogramming in ccRCC, making it a promising target for therapeutic intervention.

## Results

### MRPL12 is acetylated at Lysine 163

**Fig. 1:**
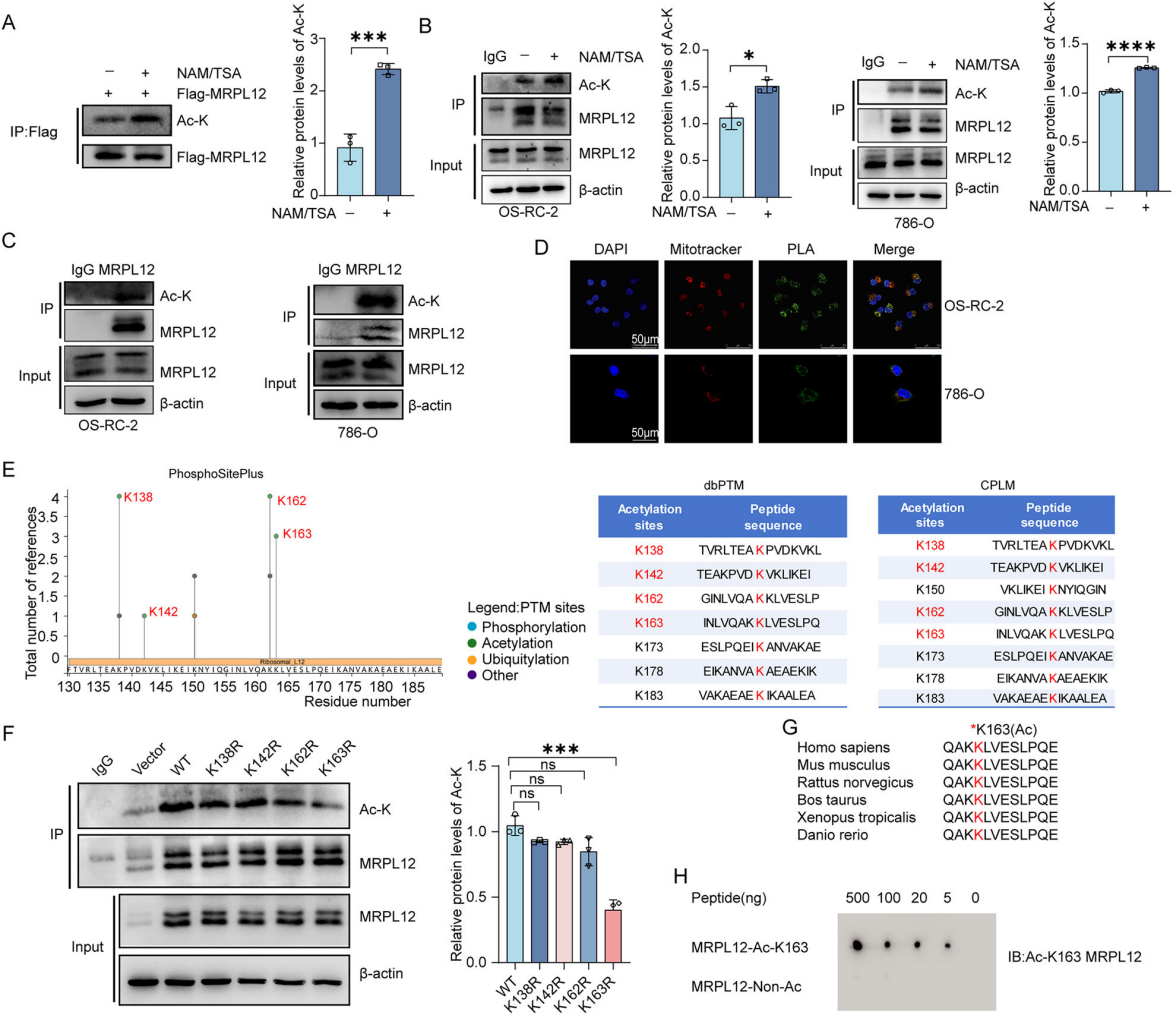
MRPL12 is acetylated at Lysine 163. **A** Immunoblot (IB) analysis of Flag-MRPL12 acetylation in HEK293T cells stably expressing Flag-MRPL12, following treatment with deacetylase inhibitors TSA (5 μM, 16 h) and NAM (10 mM, 6 h). Acetylation of MRPL12 was detected using an anti-acetylated lysine antibody. **B** IB analysis of acetylated MRPL12 in OS-RC-2 and 786-O cells, treated with TSA (5 μM, 16 h) and NAM (10 mM, 6 h) to assess endogenous acetylation levels. **C** Immunoprecipitation (IP) confirming MRPL12 acetylation in OS-RC-2 and 786-O cells. **D** Duolink assay verifying MRPL12 acetylation in OS-RC-2 and 786-O cells. Scale bar = 50 µm. **E** Identification of MRPL12 lysine acetylation modification sites obtained from PhosphoSitePlus, dbPTM and CPLM. **F** IB analysis of MRPL12 acetylation in OS-RC-2 cells transfected with wild-type (WT) or mutant forms of MRPL12. **G** Alignment of amino acid sequences surrounding Lys163 in MRPL12 across different species. **H** Dot blot assay confirming the specificity of the site-specific anti–MRPL12 K163 acetylation antibody. Significance levels: ns, *P* > 0.05; *P < 0.05; ***P < 0.001; ****P < 0.0001.

Acetylation is crucial for regulating mitochondrial protein function. However, it remains unreported whether MRPL12, as a newly identified regulator of mitochondrial biogenesis, undergoes acetylation. To confirm the presence of MRPL12 acetylation, we induced ectopic expression of Flag-MRPL12 in HEK293T cells and assessed MRPL12 acetylation levels using an anti-acetylated lysine antibody. We observed that MRPL12 acetylation increased following treatment with nicotinamide (NAM), an inhibitor of the SIRT family of deacetylases, and trichostatin A (TSA), an inhibitor of histone deacetylases HDAC I, II, and IV (Fig. 1A). Additionally, we treated OS-RC-2 and 786-O cells with NAM and TSA to evaluate the pan-acetylation levels of endogenous MRPL12, confirming the acetylation of endogenous MRPL12 (Fig. 1B and C).

Using a proximity ligation assay (PLA) (Fig. 1D), we further demonstrated MRPL12 acetylation. The PhosphoSitePlus database (https://www.phosphosite.org/) predicted four potential acetylation sites on MRPL12, while both dbPTM and CPLM databases also identify potential acetylation sites on this protein (Fig. 1E). We consolidated the prediction results from all three databases and selected four consensus acetylation sites for subsequent investigation. To identify the specific acetylation site, we generated MRPL12 mutants in which lysines (K) 138, 142, 162, and 163 were individually mutated to arginine (R) and then examined their acetylation levels. Our results showed that mutating Lys 163 significantly reduced MRPL12 acetylation, while mutations at other lysine residues had no notable effect, indicating that Lys 163 is the primary acetylation site on MRPL12 (Fig. 1F). We found that Lys 163 of MRPL12 is highly conserved across species (Fig. 1G). We further developed an antibody specifically recognizing MRPL12 acetylation at K163 and validated its specificity using a dot blot assay (Fig. 1H).

### Decreased MRPL12 K163 acetylation is linked to poor prognosis in ccRCC

**Fig. 2:**
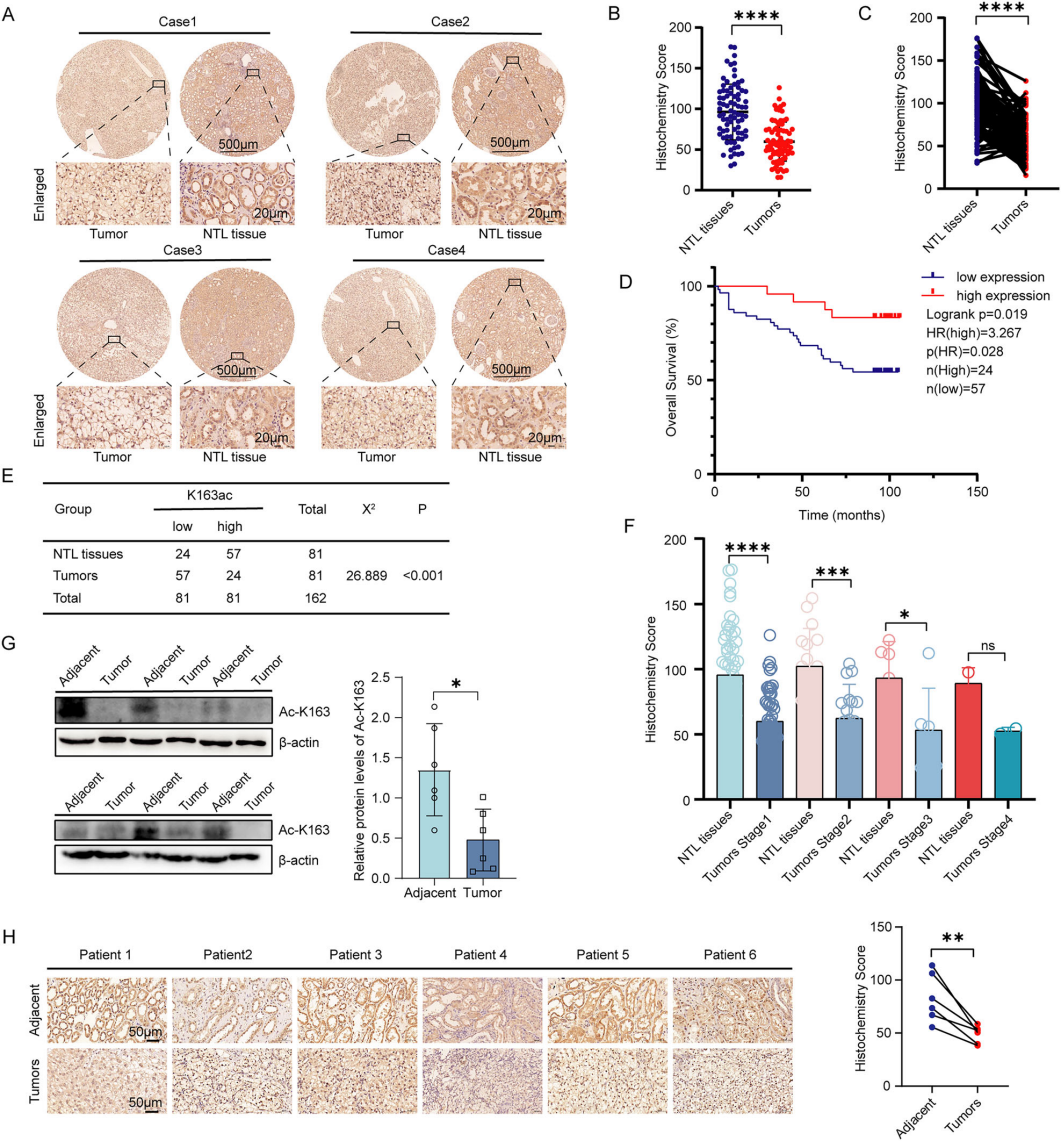
Decreased MRPL12 K163 acetylation is linked to poor prognosis in ccRCC. **A** Immunohistochemical (IHC) micrographs showing MRPL12 K163 acetylation levels in paired clinical ccRCC tumor and adjacent normal tissue microarrays (*n* = 4). Scale bars: 500 μm and 20 μm. **B** Average H-score quantifying MRPL12 acetylation levels in ccRCC and non-tumor tissue (NTL) samples. **C** H-score analysis of MRPL12 acetylation levels across 81 paired samples of ccRCC tumors and NTL tissues. **D** Kaplan–Meier survival curves comparing patient survival using the log-rank test (*n* = 81). **E** Analysis of MRPL12 K163 acetylation levels in a cohort of 81 ccRCC patients, showing a significant difference (*P* < 0.001). **F** Comparison of H-scores assessing MRPL12 acetylation levels at different disease stages. **G** Western blot (WB) analysis of MRPL12 acetylation levels in ccRCC tissues (*n* = 6). **H** IHC analysis of MRPL12 K163 acetylation levels in paired clinical ccRCC tumor and normal tissues (*n* = 6). Scale bar: 50 μm. Significance levels: ns, *P* > 0.05; *P < 0.05; **P < 0.01; ***P < 0.001; ****P < 0.0001.

To assess the clinical significance of MRPL12 K163 acetylation in ccRCC tissues, we conducted immunohistochemistry (IHC) using a specific antibody against MRPL12 K163 acetylation on tissue microarrays from 90 ccRCC patients, as well as paired ccRCC and adjacent normal tissue (NTL) samples from 81 patients. Representative tissue images are shown in Fig. 2A. The average H-score for MRPL12 acetylation in ccRCC tissues was significantly lower compared to NTL tissues (Fig. 2B). Paired H-scores also illustrated the reduced level of MRPL12 K163 acetylation in ccRCC tissues relative to their corresponding normal tissues (Fig. 2C).

When analyzing MRPL12 K163 acetylation levels in ccRCC cases, patients were divided into high and low expression groups based on the median H-score. Among the 81 ccRCC patients, those with lower MRPL12 K163 acetylation levels demonstrated significantly poorer overall survival compared to patients with higher acetylation levels (Fig. 2D). Statistical analysis confirmed that MRPL12 K163 acetylation was notably reduced in ccRCC tissues (*P* < 0.001) (Fig. 2E). Additionally, MRPL12 K163 acetylation levels were compared across ccRCC stages 1, 2, 3, and 4 (Fig. 2F). Further confirmation was obtained by examining six pairs of ccRCC tissues and their adjacent normal tissues, showing consistently lower MRPL12 K163 acetylation in ccRCC tissues (Fig. 2G and H). These results from ccRCC clinical samples indicate that low levels of MRPL12 K163 acetylation are associated with poor prognosis in ccRCC.

### MRPL12 K163 acetylation inhibits ccRCC progression

**Fig. 3:**
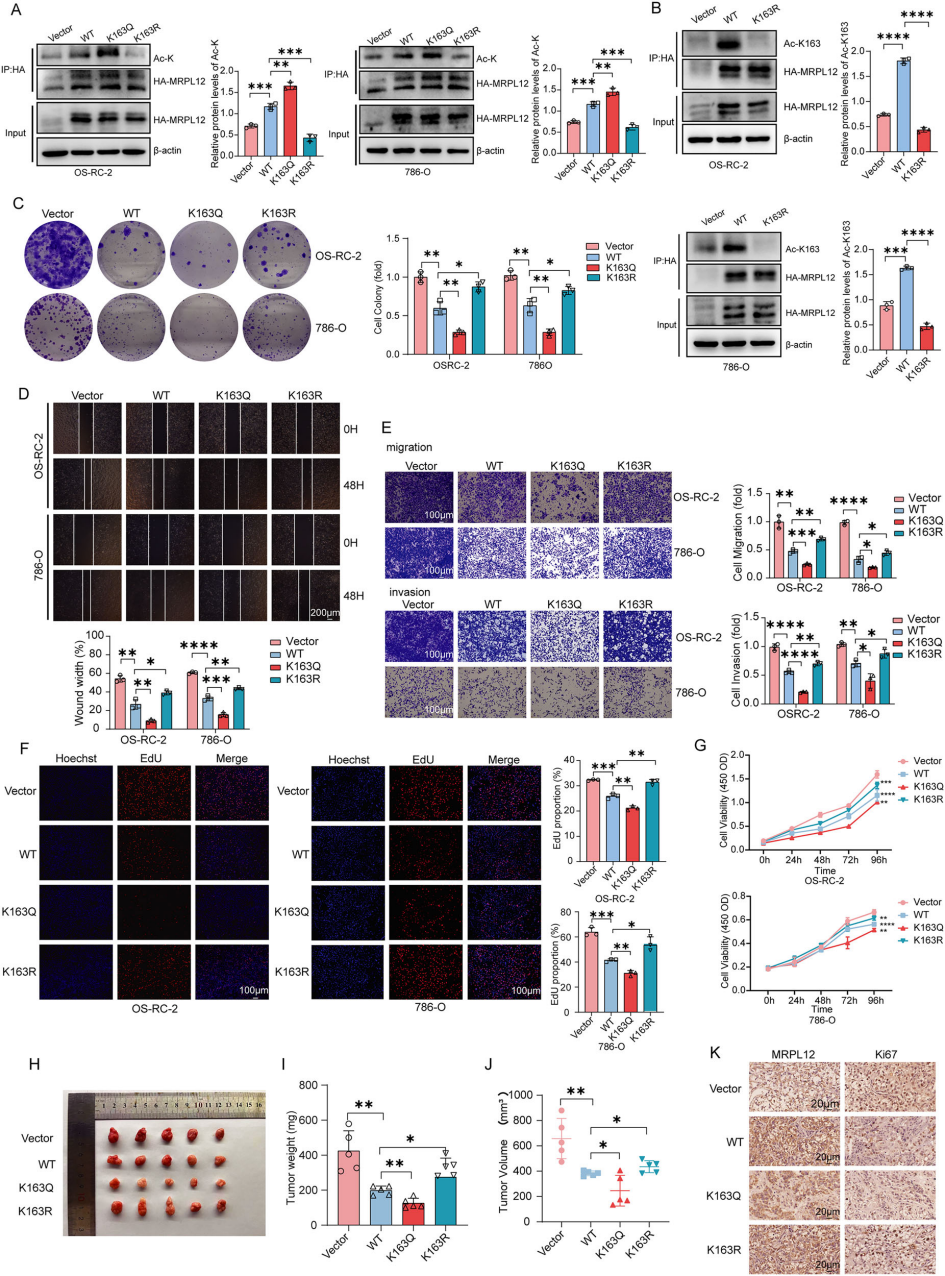
MRPL12 K163 acetylation inhibits ccRCC progression. **A** Wild-type (WT) MRPL12-HA and its K163R and K163Q mutants were transfected using lentivirus, and the acetylation of both WT and mutant MRPL12-HA proteins was detected with pan-acetylated lysine antibodies. **B** Wild-type (WT) MRPL12-HA and its K163R mutant were transfected using lentivirus, and the acetylation of both WT and mutant MRPL12-HA proteins was detected with acetylation site-specific antibodies. **C** Effects of transfecting the MRPL12 K163 mutant lentivirus on colony formation. **D** Effects of MRPL12 K163 mutant lentivirus transfection on wound healing in OS-RC-2 and 786-O cells. Scale bars: 200 μm. **E** Effects of MRPL12 K163 mutant lentivirus transfection on migration and invasion in OS-RC-2 and 786-O cells.Scale bars: 100 μm. **F** Effects of MRPL12 K163 mutant lentivirus transfection on EdU analyses. Scale bars: 100 μm. **G** Cell proliferation ability in OS-RC-2 and 786-O cells measured by Cell Counting Kit-8. **H** In vivo tumorigenesis in OS-RC-2 cells stably expressing wild-type or mutant MRPL12, assessed in a xenograft model (*n* = 5 mice per group). **I** Weights of xenograft tumors were analyzed (*n* = 5 mice per group). **J** Volumes of xenograft tumors were measured and analyzed (*n* = 5 mice per group). **K** IHC analysis on sections of xenograft tumors in nude mice to evaluate Ki-67 expression. Scale bars: 20 μm. Significance levels: *P < 0.05; **P < 0.01; ***P < 0.001; ****P < 0.0001.

To investigate the role of MRPL12 acetylation in ccRCC progression, we generated stable cell lines overexpressing MRPL12 wild-type (WT) and K163Q/K163R mutants (Fig. 3A). The K163Q mutant, where lysine 163 is replaced by glutamine, mimics lysine acetylation, while the K163R mutant, with lysine 163 replaced by arginine, serves as a non-acetylation simulation. Immunoprecipitation assays demonstrated that the K163Q mutation markedly enhanced MRPL12 acetylation, whereas the K163R mutation substantially diminished it (Fig. 3A). Furthermore, site-specific immunoblotting with an anti-acetyl-K163 antibody confirmed reduced acetylation at K163 in MRPL12 following the K163R mutation (Fig. 3B).

We next examined the impact of MRPL12 acetylation on cell proliferation. Colony formation assays (Fig. 3C) and cell viability assays (Fig. 3F and G) revealed that cells expressing the acetylation-mimic K163Q mutant had significantly reduced proliferation compared to MRPL12-WT cells, whereas the K163R mutant enhanced cell proliferation. To assess the effect of acetylation on ccRCC cell migration, we conducted wound healing and transwell assays. The K163Q mutant exhibited a more pronounced inhibitory effect on cell migration than MRPL12-WT in the wound healing assay, and transwell assays further demonstrated that the K163Q mutant significantly reduced cell migration and invasion compared to MRPL12-WT cells. In contrast, the K163R mutant promoted cell migration (Fig. 3D and E).

In vivo experiments were conducted to assess the impact of MRPL12 acetylation on ccRCC tumorigenicity. MRPL12 acetylation at K163 significantly inhibited tumor formation in nude mice, resulting in smaller tumors (Fig. 3H–J). Immunohistochemical analyses of xenografts confirmed corresponding changes in MRPL12 and Ki-67 expression levels (Fig. 3K). In summary, these findings suggest that MRPL12 K163 acetylation is a crucial factor in ccRCC tumorigenesis and progression, as demonstrated by both in vitro and in vivo studies.

### MRPL12 K163 acetylation is involved in driving metabolic reprogramming

**Fig. 4:**
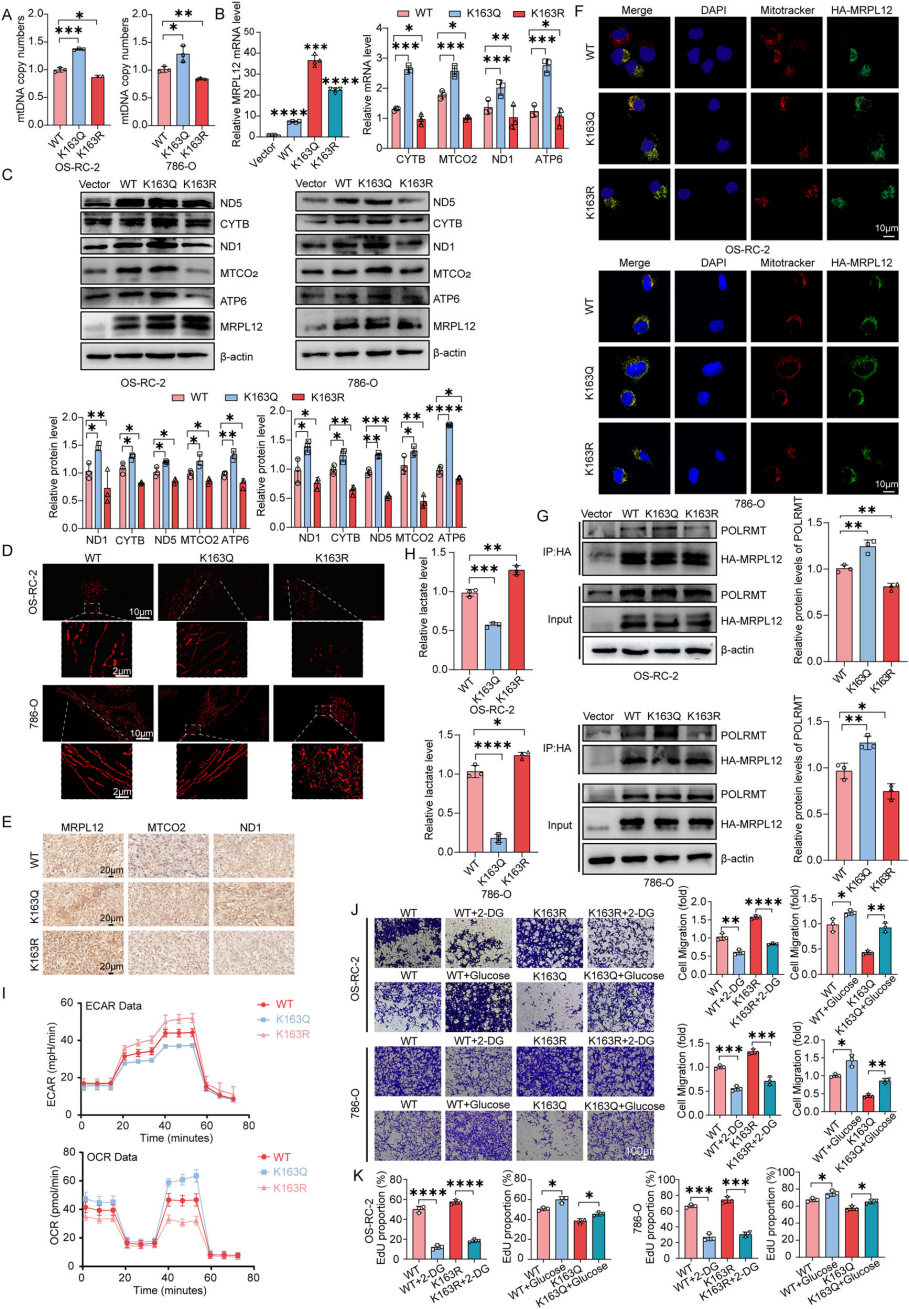
MRPL12 K163 acetylation is involved in driving metabolic reprogramming. **A** Mitochondrial DNA (mtDNA) copy number analysis in OS-RC-2 and 786-O cells. **B** mRNA expression levels of genes encoding subunits of the respiratory chain complexes. **C** Protein expression levels of genes encoding subunits of the respiratory chain complexes. **D** Morphological changes in mitochondria observed using ultra-high resolution microscope 3D imaging system. Scale bars: 2 μm, 10 μm. **E** IHC analysis of xenograft tumor sections from nude mice to evaluate the expression of genes associated with respiratory chain complexes. Scale bars: 20 μm. **F** Co-localization of MRPL12 with mitochondria assessed by immunofluorescence staining. Scale bars: 10 μm. **G** Interaction between MRPL12 and POLRMT verified by immunoblot (IB) analysis. **H** Lactic acid content in OS-RC-2 and 786-O cells transfected with wild-type and mutant MRPL12 lentivirus. **I** Extracellular acidification rate (ECAR) and oxygen consumption rate (OCR) in OS-RC-2 cells following transfection with wild-type and mutant MRPL12 lentivirus. **J** Effects of 2-DG and glucose on cell migration in OS-RC-2 and 786-O cells. Scale bars: 100 μm. **K** Effects of 2-DG and glucose on EdU analysis in OS-RC-2 and 786-O cells. Significance levels: *P < 0.05; **P < 0.01; ***P < 0.001; ****P < 0.0001.

Previous literature and our research indicate that MRPL12 serves as a critical regulatory factor in mitochondrial biogenesis and cellular metabolism. We hypothesized that the tumor-suppressive function of MRPL12 acetylation in ccRCC may be linked to metabolic reprogramming. To investigate this, we assessed the impact of MRPL12 acetylation on mtDNA, essential for maintaining normal mitochondrial respiratory function. Our results demonstrated that mtDNA copy number decreased with the MRPL12 K163R mutation, whereas it increased with K163Q mutant overexpression (Fig. 4A). To further examine the effect of MRPL12 K163 acetylation on the expression of mtDNA-encoded respiratory chain complex subunits, we evaluated ND1, ND5, MTCO2, CYTB, and ATP6 protein and mRNA levels in ccRCC cells. The K163R mutant led to a downregulation of these genes expression, while the K163Q mutant enhanced their expression (Fig. 4B and C). Additionally, mitochondrial labeling with MitoTracker Red and ultra-high resolution microscope 3D imaging revealed that mitochondria in K163R mutant cells appeared fragmented and swollen (Fig. 4D). Immunohistochemistry on xenograft sections derived from K163Q and K163R mutant overexpression in OS-RC-2 cells showed alterations in mtDNA-encoded respiratory chain complex subunits, consistent with observations in ccRCC cells (Fig. 4E). To elucidate the mechanism by which MRPL12 K163 acetylation regulates mitochondrial function, we examined MRPL12 localization in labeled mitochondria and found no significant alterations between K163Q and K163R mutant cells (Fig. 4F). MRPL12 has been shown to stabilize POLRMT and directly activate mitochondrial transcription by binding to POLRM [15–17]. We verified that MRPL12 acetylation increases its binding to POLRMT, promoting mtDNA transcription (Fig. 4G). Moreover, as a glycolytic product, lactate production was reduced in K163Q mutant cells (Fig. 4H). We also assessed the extracellular acidification rate (ECAR) as an indicator of glycolysis and the oxygen consumption rate (OCR) as an indicator of oxidative phosphorylation. The results indicated that K163Q mutant reduced ECAR and increased OCR (Fig. 4I). The above findings suggest that MRPL12 K163 acetylation drives metabolic reprogramming in ccRCC cells.

Notably, we found that the proliferative and migratory effects induced by K163R mutant overexpression in ccRCC cells were effectively suppressed by treatment with a glycolysis inhibitor, suggesting that the malignant phenotype resulting from MRPL12 K163R overexpression is facilitated through enhanced glycolysis (Fig. 4J, K, and Supplementary Fig. 1A). Additionally, EdU and Transwell migration assays revealed that glucose-treated K163Q-overexpressing cells exhibited significantly increased migration and proliferation, indicating that the suppression of the malignant phenotype associated with MRPL12 K163Q overexpression can be partially reversed by promoting glycolysis (Fig. 4J, K, and Supplementary Fig. 1A). Collectively, these results demonstrate that MRPL12 K163 acetylation inhibits glycolysis in ccRCC cells, thereby affecting ccRCC progression. In conclusion, MRPL12 K163 acetylation may suppress ccRCC progression by serving as a critical modulator of mitochondrial metabolism.

### Deacetylase SIRT5 deacetylates MRPL12 at K163

**Fig. 5:**
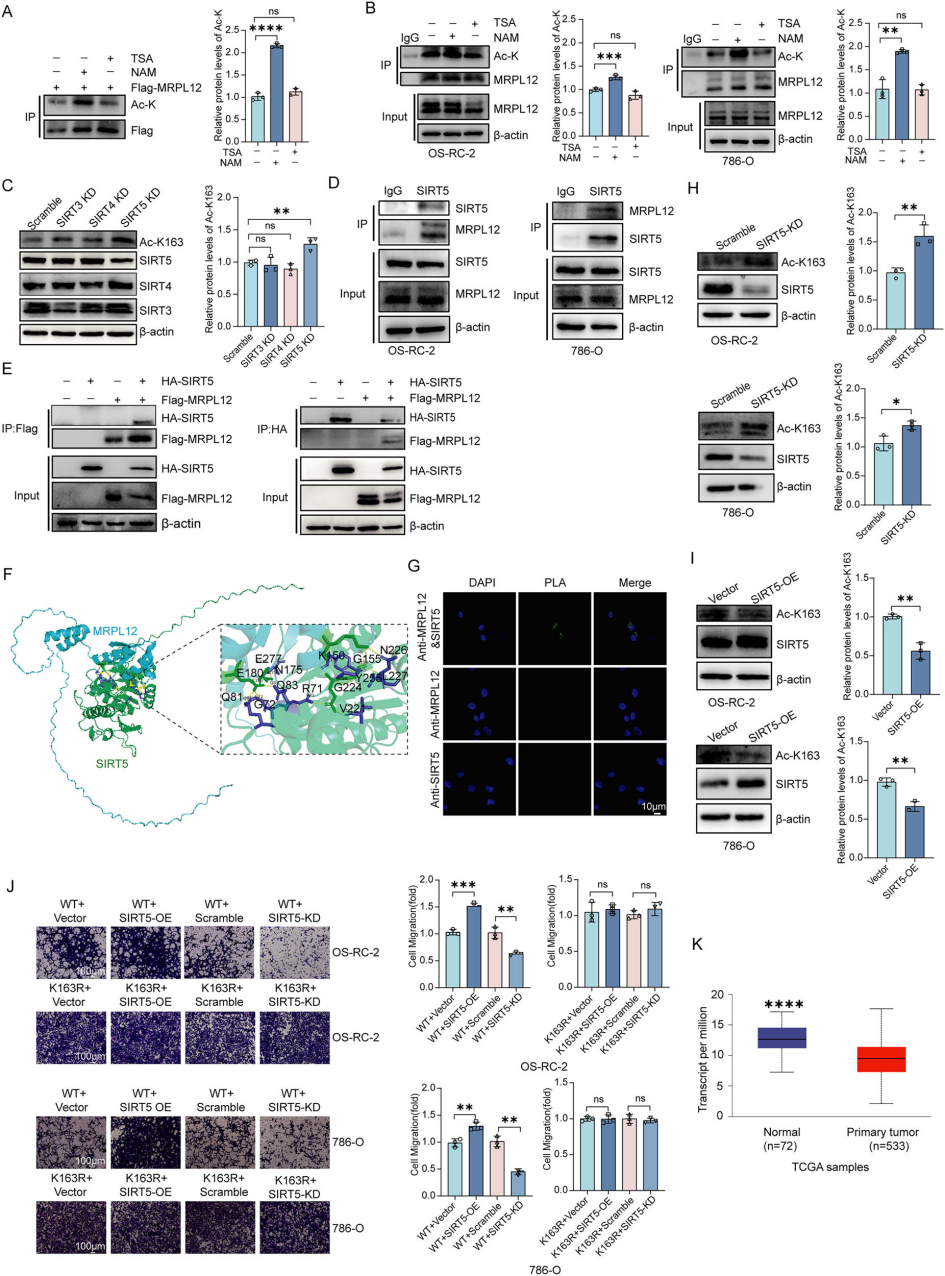
Deacetylase SIRT5 deacetylates MRPL12 at K163. **A** Immunoblot (IB) analysis of acetylation, stably expressed Flag-MRPL12 in HEK293T cells treated with TSA (5 μM for 16 h) and NAM (10 mM for 6 h). **B** IB analysis of acetylation, stably expressed MRPL12 in OS-RC-2 and 786-O cells treated with TSA (5 μM for 16 h) and NAM (10 mM for 6 h). **C** Analysis of MRPL12 K163 acetylation levels in OS-RC-2 cells transfected with siRNA targeting SIRT deacetylases. **D** Endogenous interaction between MRPL12 and SIRT5 in OS-RC-2 and 786-O cells, determined by immunoprecipitation (IP) and IB analysis. **E** HEK293T cells transfected with Flag-MRPL12 and HA-SIRT5 as indicated. Interactions between MRPL12 and SIRT5 were determined by co-IP and IB analysis. **F** AlphaFold 3 simulation showing the predicted interaction between MRPL12 and SIRT5. **G** Duolink proximity ligation assay demonstrating the interaction between MRPL12 and SIRT5 in OS-RC-2 cells. Scale bar: 10 μm, *n* = 3 per group. **H** Endogenous MRPL12 K163 acetylation increases upon SIRT5 silencing. **I** Endogenous MRPL12 K163 acetylation decreases with SIRT5 overexpression. **J** Effect of SIRT5 knockdown and overexpression on cell migration in OS-RC-2 cells. Scale bar: 100 μm. **K** SIRT5 expression levels in clear cell renal cell carcinoma (ccRCC) tissues compared with normal tissue (NTL) tissues, as shown in the UALCAN database, indicating reduced expression in ccRCC. Significance levels: ns, *P* > 0.05; *P < 0.05; **P < 0.01; ***P < 0.001; ****P < 0.0001.

To identify deacetylases responsible for MRPL12 deacetylation, we induced ectopic expression of Flag-MRPL12 in 293 T and assessed acetylation levels using an anti-acetylated antibody. Treatment with nicotinamide (NAM), an inhibitor of the SIRT family of deacetylases, significantly enhanced MRPL12 acetylation (Fig. 5A). However, the TSA did not significantly affect the acetylation level of MRPL12. Similarly, treatment with NAM, but not TSA, in OS-RC-2 and 786-O cells resulted in increased endogenous acetylation of MRPL12 (Fig. 5B). Given MRPL12’s mitochondrial localization, we selected three mitochondrial deacetylases for further investigation. Notably, SIRT5 knockdown, but not the other two deacetylases, markedly promoted MRPL12 K163 acetylation (Fig. 5C). We also confirmed the interaction between SIRT5 and endogenous MRPL12 through co-immunoprecipitation (Fig. 5D) and further verified this interaction by ectopically expressing Flag-MRPL12 and HA-SIRT5 in HEK293T cells (Fig. 5E).

Using Alphafold3, we predicted the interaction model between MRPL12 and SIRT5, including potential amino acid contact sites (Fig. 5F). A PLA assay further confirmed the interaction of MRPL12 and SIRT5 (Fig. 5G). SIRT5 knockdown significantly increased endogenous MRPL12 K163 acetylation, whereas SIRT5 overexpression decreased acetylation. (Fig. 5H and I). These results collectively indicate that SIRT5 is a primary deacetylase of MRPL12 at K163.

In functional assays, SIRT5 knockdown suppressed migration and proliferation of ccRCC cells, even in the presence of MRPL12-WT overexpression. Conversely, SIRT5 overexpression promoted these malignant phenotypes (Fig. 5J). However, neither SIRT5 knockdown nor overexpression significantly affected migration (Fig. 5J) or proliferation (Supplementary Fig. 1B) in the K163R mutant group, suggesting that SIRT5 specifically recognizes and deacetylates the K163 site on MRPL12, thereby promoting the malignant phenotype of ccRCC cells. These findings suggest that SIRT5 interacts with and mediates the deacetylation of MRPL12, positioning SIRT5 as a potential deacetylase for MRPL12.

Finally, we analyzed SIRT5 expression in renal cancer tissues and observed that it was significantly downregulated (Fig. 5K). Considering that the acetylation level of the MRPL12 K163 site is significantly lower in ccRCC tissues compared to adjacent normal tissues, it is likely that the reduced SIRT5-mediated acetylation of the MRPL12 K163 site in ccRCC has little effect on tumour progression, but rather by other regulatory mechanisms.

### TIP60 involves in MRPL12 K163 acetylation in ccRCC

**Fig. 6:**
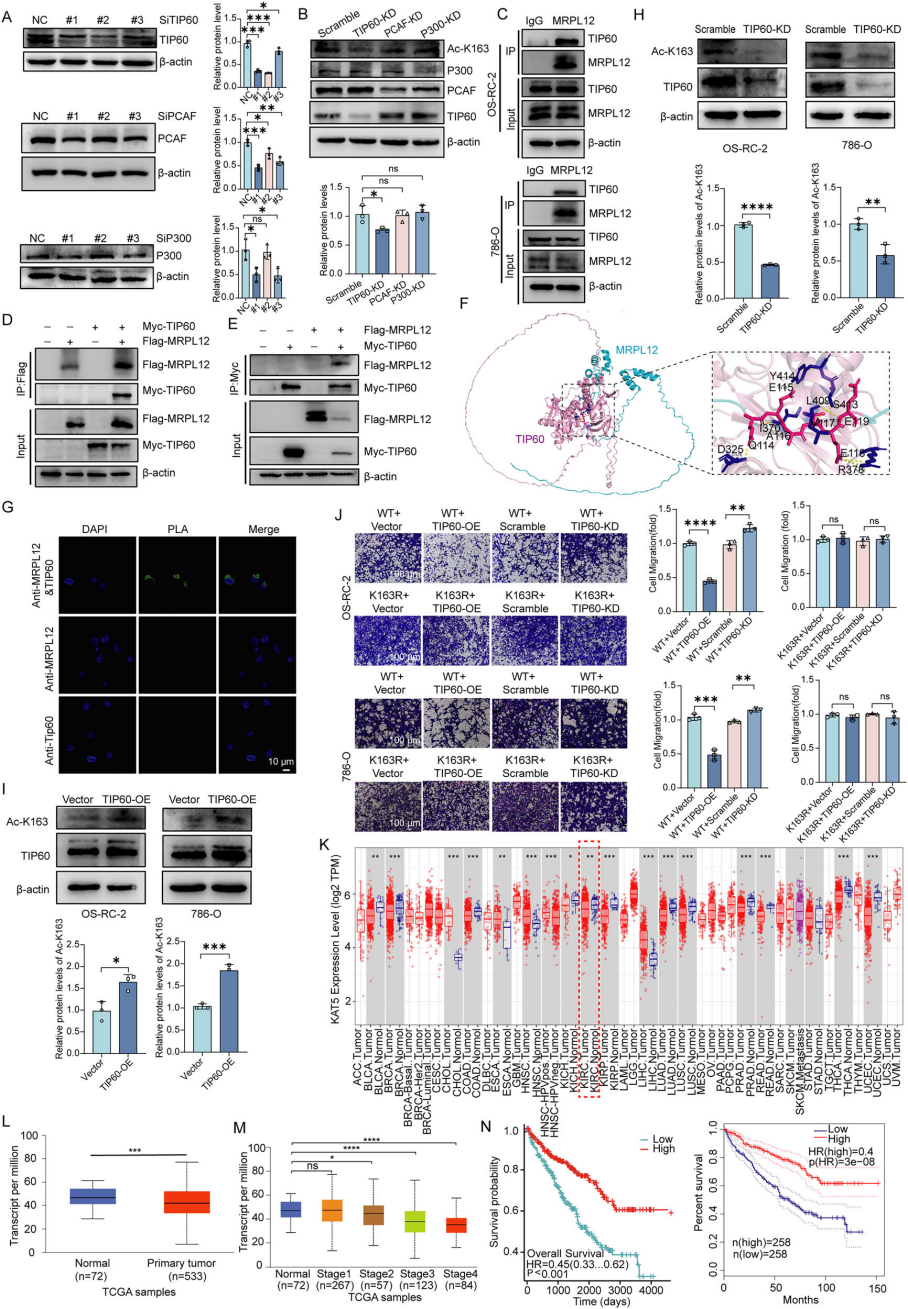
TIP60 involves in MRPL12 K163 Acetylation in ccRCC. **A** TIP60, PCAF, and P300 siRNA were transfected into HEK293T cells, and their knockdown efficiencies were assessed by Western blot (WB) analysis. **B** MRPL12 K163 acetylation levels in OS-RC-2 cells were analyzed following transfection with siRNA targeting TIP60, PCAF, and P300. **C** Endogenous interactions between MRPL12 and TIP60 in OS-RC-2 and 786-O cells were determined by immunoprecipitation (IP) and immunoblot (IB) analysis. **D, E** HEK293T cells were transfected with Flag-MRPL12 and Myc-TIP60 as indicated. Interactions between MRPL12 and TIP60 were assessed by co-IP and IB analysis. **F** AlphaFold 3 simulation illustrating the predicted interaction between MRPL12 and TIP60. **G** Interaction between MRPL12 and TIP60 in OS-RC-2 cells detected by Duolink proximity ligation assay. Scale bar: 10 μm, *n* = 3 per group. **H** Silencing TIP60 decreased endogenous K163 acetylation of MRPL12. **I** TIP60 overexpression increased endogenous K163 acetylation of MRPL12. **J** Effect of TIP60 knockdown and overexpression on cell migration. Scale bar: 100 μm. **K** Regulation of TIP60/KAT5 expression across various tumor types as shown in The Cancer Genome Atlas (TCGA) Database. **L** Comparison of TIP60/KAT5 expression between ccRCC tissues and normal tissue (NTL), showing reduced expression in ccRCC from the UALCAN database. **M** Significant correlation between TIP60/KAT5 expression and ccRCC staging identified in the UALCAN database. **N** Survival analysis evaluating overall survival of ccRCC patients, conducted using the GEPIA and Xiantao databases. Significance levels: ns, *P* > 0.05; *P < 0.05; **P < 0.01; ***P < 0.001; ****P < 0.0001.

To identify the enzyme responsible for MRPL12 K163 acetylation, we investigated three acetyltransferases—p300, TIP60, and PACF—and confirmed their knockdown efficiencies (Fig. 6A). Notably, TIP60 knockdown, but not the other two acetyltransferases, significantly reduced MRPL12 K163 acetylation (Fig. 6B). We further validated the interaction between endogenous MRPL12 and TIP60 through co-immunoprecipitation in OS-RC-2 and 786-O cells (Fig. 6C). Additionally, we ectopically expressed Flag-MRPL12 and MYC-TIP60 in HEK293T cells to verify this interaction (Fig. 6D and E).

Using Alphafold3, we predicted the structural interaction between MRPL12 and TIP60, including potential amino acid contact sites (Fig. 6F). This interaction was further demonstrated by PLA assay (Fig. 6G). TIP60 knockdown significantly reduced endogenous MRPL12 K163 acetylation, while TIP60 overexpression enhanced it (Fig. 6H and I). Collectively, these findings indicate that TIP60 acts as the acetyltransferase for MRPL12 at K163.

Transwell migration and EdU assays demonstrated that TIP60 knockdown promoted, while TIP60 overexpression suppressed, migration and proliferation in ccRCC cells overexpressing WT MRPL12. This finding suggests that TIP60 influences the malignant phenotype of ccRCC cells through interactions with WT MRPL12. Notably, neither knockdown nor overexpression of TIP60 significantly affected migration (Fig. 6J) or proliferation (Supplementary Fig. 1C) in cells overexpressing the K163R mutant, indicating that TIP60’s effects are mediated specifically through recognition of the MRPL12 K163 site. These results suggest that TIP60 specifically mediates MRPL12 K163 acetylation, thereby contributing to the regulation of the malignant phenotype in ccRCC cells.

Protein expression data from the UALCAN database confirmed significantly lower levels of TIP60 in ccRCC tissues compared to adjacent normal tissues (Fig. 6K and L). Furthermore, TIP60 expression was correlated with tumor stage (Fig. 6M), and lower TIP60 mRNA levels were associated with shorter overall survival in ccRCC patients, as indicated by analyses from the GEPIA and Xiantao databases (Fig. 6N). In conclusion, TIP60 is a primary factor responsible for the significant reduction in MRPL12 K163 acetylation levels in ccRCC and plays a role in regulating the malignant phenotype of ccRCC cells.

## Discussion

In this study, we established a novel link between MRPL12 K163 acetylation-induced metabolic reprogramming and the progression of ccRCC. Our previous studies have demonstrated that MRPL12 is implicated in various metabolic diseases, including diabetic nephropathy, acute kidney injury, liver cancer, and lung cancer, through its regulation of mitochondrial biosynthesis [18–24]. These findings underscore the significance of MRPL12 in the initiation and progression of diverse pathological conditions [32–34]. In the current study, we observed low levels of MRPL12 K163 acetylation in ccRCC and identified a strong correlation between MRPL12 K163 acetylation levels, tumor stages, and prognosis in ccRCC patients. However, we unexpectedly observed that the difference in MRPL12 K163 acetylation between tumor and normal tissues (NTL) gradually decreased from stage I to stage IV. We speculate that this may be due to the limited sample size in stages III and IV, highlighting the need for a larger cohort of stage III and IV cases to validate this trend. By functionally characterizing MRPL12 acetylation through stable MRPL12-WT, K163Q, and K163R mutant overexpression models in vitro and in vivo, we found that MRPL12 K163 acetylation suppresses cell proliferation and restricts migration and invasion.

Mitochondrial dysfunction and enhanced glycolysis are hallmarks of ccRCC. While glycolysis has been extensively studied, the role of mitochondrial contributions to ccRCC progression remains less understood [35]. Here, we found that MRPL12 K163 acetylation is involved in mitochondrial dysfunction in ccRCC. Following MRPL12 deacetylation, we observed abnormal mitochondrial structural changes in ccRCC cells. Furthermore, mitochondrial DNA copy number increased with MRPL12 K163 acetylation, and the expression of mitochondrial DNA-encoded respiratory chain complex subunits was promoted by MRPL12 K163 acetylation. In addition, we observed that although the mRNA levels of MRPL12 wild-type (WT) and its mutants differ, the protein expression levels remain consistent, indicating that further investigation into the mechanisms underlying the discrepancies between mRNA and protein levels in different MRPL12 mutants is needed. These findings indicate that MRPL12 K163 acetylation may play an cancer inhibition role in ccRCC by inhibiting glycolysis.

Metabolic reprogramming is a common feature in various cancer types [36, 37]. Shifts between glycolysis and OXPHOS under hypoxic conditions provide metabolic flexibility that benefits cancer cells. The Warburg effect, characterized by aerobic glycolysis, is a hallmark of most cancers and is especially pronounced in ccRCC [38, 39]. Reduced glycolytic activity in ccRCC may be associated with increased OXPHOS and the presence of mitochondrial mutations or elevated mtDNA content necessary for normal respiratory function. MRPL12 has been shown to regulate the stability of POLRMT, with MRPL12 directly binding to POLRMT and activating mitochondrial transcription. In vitro studies have determined that IMT1 and IMT1B non-competitively inhibit POLRMT, resulting in conformational changes that impede substrate binding and transcription in a dose-dependent manner [40]. POLRMT mutations disrupt mitochondrial transcription and are linked to various diseases [41]. Our study confirmed that MRPL12 acetylation enhances its interaction with POLRMT, thereby promoting mtDNA transcription. We also observed that MRPL12 acetylation is involved in metabolic reprogramming, as MRPL12 K163-acetylated ccRCC cells showed reduced ECAR and lactate production. Glycolysis inhibition suppressed MRPL12 K163R-overexpressing ccRCC cell proliferation and migration, suggesting that MRPL12 K163 deacetylation may promote oncogenesis by fostering glycolysis and inhibiting mitochondrial biosynthesis.

To identify factors that regulate MRPL12 acetylation, we screened acetyltransferases and deacetylases. Our findings indicate that TIP60 serves as an acetyltransferase that increases MRPL12 K163 acetylation and inhibits ccRCC migration. Furthermore, TIP60 expression correlated with tumor stage, and analysis of ccRCC patient data from the TCGA database showed that lower TIP60 (KAT5) mRNA levels are associated with poorer survival. SIRT5 acts as a deacetylase that reduces MRPL12 K163 acetylation, but its role in ccRCC may be less significant than that of acetyltransferases, as it may primarily mediate MRPL12 acetylation in normal contexts. SIRT5 has also been reported to desuccinylate SDHA, promoting ccRCC tumorigenesis [42]. These findings suggest that targeting MRPL12 acetyltransferases may have therapeutic potential in ccRCC.

In conclusion, our study reveals that MRPL12 K163 acetylation drives metabolic shifts from glycolysis to oxidative phosphorylation, inhibiting ccRCC development. While the specific mechanisms underlying this metabolic switch remain undefined, our findings provide new insights into MRPL12 acetylation and lay a foundation for future investigations into the therapeutic potential of modulating MRPL12 acetylation in ccRCC.

## Methods

### Cell culture

OS-RC-2 and 786-O cells were procured from Haixing Biosciences and cultured in RPMI-1640 medium (Gibco, Grand Island, NY) with 10% fetal bovine serum (FBS, Cas9X, China), while 293 T cells were cultured in DMEM (Gibco, Grand Island, NY) with 10% FBS. All cells were maintained in a humidified atmosphere at 37 °C with 5% CO₂. These cells were authenticated using short tandem repeat (STR) profiling, and no mycoplasma contamination was found.

### Patients and specimens

Human ccRCC tissues and adjacent noncancerous renal tissues were collected from Provincial Hospital of Shandong First Medical University. A tissue microarray, consisting of specimens from 90 ccRCC patients, was obtained from Shanghai Outdo Biotech Co., Ltd. (Shanghai, China).

### Transfection, virus infection, and treatments

Small interfering RNAs (siRNAs) targeting SIRT3, SIRT4, SIRT5, TIP60, PCAF, P300, and a non-targeting negative control siRNA were purchased from RiboBio (China). OS-RC-2 and 786-O cells were transfected with 50 nM siRNA using RNA TransMate (Sangon Biotech) for 48 h. For lentiviral infection, target cells were incubated in a lentivirus-containing medium with polybrene (10 μg/mL) for 48 h, followed by puromycin selection. Overexpression plasmids and control empty vectors were constructed by Biosune Biotechnology (Shanghai, China) and transfected into cells in 6-well plates using Lipofectamine 3000 (Invitrogen, Shanghai, China). For deacetylase inhibitor treatments, TSA (5 μM) and NAM (10 mM) were added to the culture medium for 16 and 6 h, respectively, before harvesting. Glycolysis inhibitor 2-DG (10 mM) and glucose (10 mM) were added to the culture medium for 48 h.

### Immunoblot and immunoprecipitation (IP) analysis

Cells were lysed in IP lysis buffer with protease inhibitors, centrifuged at 12,000 rpm for 15 min, and the supernatant was collected as input. Lysates were incubated with 3 µg of primary antibody or control rabbit IgG coupled to 50 µL of Protein A/G beads overnight. Protein concentration was assessed using a BCA Protein Assay Kit. Following incubation, beads were washed, antigens were eluted, and samples were analyzed by Western blot. Proteins were separated by SDS-PAGE, transferred to PVDF membranes, and detected with primary antibody and HRP-conjugated secondary antibodies (Invitrogen, Shanghai, China).

### Western blot analysis

Cells and kidney tissues were lysed in RIPA buffer (Beyotime, P0013B) with 1% phenylmethylsulfonyl fluoride (PMSF, Solarbio, P0100). After centrifugation at 12,000 rpm for 30 min, protein concentrations were determined using a BCA Protein Assay Kit (Beyotime, P0011). Lysates were diluted in 4x SDS-PAGE loading buffer (Solarbio, P1015) and boiled at 95 °C for 5 min. Proteins were separated by SDS-PAGE, transferred to PVDF membranes (Millipore), blocked with 0.5% skim milk, and incubated with primary antibodies. Detection was carried out using HRP-conjugated anti-rabbit or anti-mouse IgG antibodies and chemiluminescent reagents (ECL, Millipore). Antibodies used included anti–acetylated lysine (PTM-105RM), anti-MRPL12 (Proteintech, 14795-1-AP), anti-actin (Servicebio, GB15003-100), anti-SIRT3 (Proteintech, 10099-1-AP), anti-SIRT4 (Thermo Fisher Scientific, PA5-81259), anti-SIRT5 (Proteintech, 15122-1-AP), anti-Flag (Proteintech, 20543-1-AP), anti-HA (Proteintech, 51064-2-AP), anti-Myc (Proteintech,16286-1-AP), anti-CYTB (Proteintech,55090-1-AP), anti-MTCO2 (Proteintech, 55070-1-AP), anti-ND1 (Proteintech, 19703-1-AP), anti-ND5 (Proteintech, 55410-1-AP), anti-ATP6 (ImmunoWay, YN0468), anti-TIP60 (Proteintech, 10827-1-AP), anti-PCAF (Proteintech, 28770-1-AP), anti-P300 (Proteintech, 20695-1-AP), and anti-acetyl-K163 MRPL12 (Shanghai Qiangyao Biological Technology Co., Ltd).

### Duolink proximity ligation assay (PLA)

OS-RC-2 and 786-O cells were incubated in RPMI-1640 with 100 nM MitoTracker Red CMXRos (Invitrogen, M7512) for 30 min and washed three times with PBS. Duolink® Blocking Solution was added to each sample and incubated for 60 min at 37 °C. Primary antibodies were diluted in Duolink® Antibody Diluent and incubated overnight at 4 °C. Slides were washed with Buffer A, then incubated with PLA Probe Solution for 1 h at 37 °C. Ligase was added to Ligation Buffer, and slides were incubated for 30 min at 37 °C. Amplification Buffer was then prepared with Polymerase, and slides were incubated for 100 min at 37 °C. Staining was performed with DAPI for 15 min. Images were captured using a Leica DMi8 microscope.

### Quantitative real-time PCR (qRT-PCR)

Total RNA was extracted from treated cells using TRIzol Reagent (Invitrogen, Shanghai, China) according to the manufacturer’s instructions. mRNA was reverse-transcribed to cDNA using the PrimeScript™ RT Reagent Kit (Perfect Real Time; TAKARA, Beijing, China). Quantitative real-time PCR was performed in triplicate using 0.2 mL 8-Tube PCR Strips with Attached Caps (Kirgen Bioscience, Shanghai, China) on a QuantStudio™ Real-Time PCR Instrument (Thermo Fisher Scientific, Shanghai, China). The primer sequences used were as follows:

- β-actin: F-CATGTACGTTGCTATCCAGGC; R-CTCCTTAATGTCACGCACGAT
- MRPL12: F-CCAAGGCATCAACCTCGTCC; R-AGCTTTGGCGACATTGGCTT
- ND1: F-CGATTCCGCTACGACCAACT; R-AGGTTTGAGGGGGAATGCTG
- ATP6: F-GCCACAACTAACCTCCTCGG; R-GGTAAGAAGTGGGCTAGGGC
- CYTB: F-CACTACTCACCAGACGCCTCA; R-GCGTGAAGGTAGCGGATGAT
- MT-CO2: F-AACCGTCTGAACTATCCTGCC; R-AAGATTAGTCCGCCGTAGTCG

### Mitochondrial DNA copy number quantification

Total DNA was isolated from treated cells using the FastPure Cell/Tissue DNA Isolation Mini Kit (Nanjing Vazyme Biotech Co., Ltd.). Quantitative real-time PCR (qPCR) assays were performed using the SYBR Green Premix Pro Taq HS qPCR Kit (Accurate Biology). For mtDNA quantification, the following primer sequences were used:

- D-Loop 2: F-GGCTCTCAACTCCAGCATGT; R-AGGACGAGGGAGGCTACAAT
- G6PC (nuclear control): F-CTGTCTTTGATTCCTGCCTCAT; R-GTGGCTGTGCAGACATTCAA

### Immunofluorescence staining

To label mitochondria, OS-RC-2 and 786-O cells were washed with phosphate-buffered saline (PBS) and incubated in RPMI-1640 containing 100 nM MitoTracker™ Red CMXRos (Invitrogen, M7512) for 30 min. Following incubation, cells were washed three times with PBS for further analysis.For immunofluorescence staining, cells were fixed in 4% paraformaldehyde, permeabilized with 0.5% Triton X-100, and blocked with 0.5% BSA. Cells were incubated with primary antibodies overnight at 4 °C, followed by staining with Alexa Fluor 647 Goat Anti-Rabbit IgG H&L (1:400) for 1 h and counterstaining with DAPI for 5 min. Images were captured using a Leica DMi8 microscope.

### Immunohistochemistry and scoring

Kidney samples were fixed, paraffin-embedded, and sectioned into 3 μm slices. Sections were deparaffinized with dimethylbenzene, rehydrated through a gradient of alcohol, and subjected to antigen retrieval in citric acid buffer (pH 6.0). After cooling to room temperature, sections were washed with PBS and blocked with 10% goat serum for 30 min. Primary antibodies were applied overnight at 4 °C, followed by incubation with the secondary antibody (PV-9000, Zhongshan Biotechnology Co., Beijing, China) for 1 h at 37 °C. Slides were stained with DAB, counterstained with hematoxylin, and imaged using a Pannoramic SCAN II. Immunohistochemical staining was evaluated using the histochemistry score (H-score) by QuPath software. H-scores, ranging from 0 to 300, were independently assessed by three pathologists blinded to clinicopathologic information and patient outcomes. Scores < median were classified as low expression, and scores ≥ median were classified as high expression.

### CCK-8 cell viability assays

Cells were seeded in 96-well plates (2000 cells/well) with 100 μL of medium and incubated overnight. Treatments were applied for 0, 24, 48, 72, or 96 h. At each time point, 10 μL of CCK-8 reagent (Beijing Biosynthesis Biotechnology Co., Ltd.) was added to each well. After 1 h of incubation, optical density (OD) was measured at 450 nm using a SpectraMax i3x microplate reader (USA). Experiments were performed in triplicate.

### EdU assay

The EdU assay was conducted using the EdU Cell Proliferation Kit (Beyotime, #C0075). OS-RC-2 and 786-O cells were treated with 5 μM EdU under indicated conditions for 2 h. Cells were fixed with 4% paraformaldehyde for 20 min, permeabilized with 0.3% Triton X-100 for 25 min, and incubated with the Click Reaction solution for 30 min in the dark. After three washes with PBS, cells were stained with 0.1% Hoechst 33342 for 10 min and imaged with a fluorescence microscope.

### Transwell assays

Transwell experiments were conducted in 24-well plates. Approximately 4 × 10⁴ cells/well were seeded in the upper chamber in serum-free medium, while the lower chamber contained 30% serum medium. Migration and invasion assays were conducted with and without Matrigel coating on the Transwell chambers, respectively. After migration, cells were fixed with 4% paraformaldehyde and stained with hematoxylin.

### Colony formation assay

Cells were plated at a density of 1000 cells/well in 6-well plates and grown for 14 days, with media changes every 3–4 days. Once colonies formed, they were washed with PBS, fixed with 4% paraformaldehyde, and stained with hematoxylin (Solarbio, Beijing, China).

### Seahorse assay

Oxygen consumption rate (OCR) and extracellular acidification rate (ECAR) were measured using a Seahorse XFe96 analyzer with the Seahorse XF Cell Mito Stress Test Kit and XF Glycolysis Stress Test Assay, according to the manufacturer’s instructions (Agilent Technologies, Beijing, China). Treated cells were plated in XF96 cell culture microplates and incubated at 37°C with 5% CO₂ overnight. Before the assay, the medium was changed to a pre-warmed assay medium, and cells were incubated for 1 h without CO₂. For OCR, cells were treated sequentially with oligomycin (1 μM), FCCP (1.5 μM), and rotenone/antimycin A (0.5 μM). For ECAR, cells were treated with glucose (10 mM), oligomycin (1 μM), and 2-DG (50 mM).

### Measurement of lactate production

Lactate production was measured using a Lactic Acid Assay Kit (Abbkine, #ktb1100) following the manufacturer’s protocol. Briefly, 50 μL of cell supernatant was combined with 50 μL of Lactate Assay Buffer and 50 μL of Working Reagent in a 96-well plate. The mixture was incubated at 37 °C for 30 min in the dark. Absorbance was measured at 450 nm using a SpectraMax i3x microplate reader (USA). Lactate production was normalized to the protein concentration.

### ccRCC tumor xenograft mouse model

Four- to five-week-old female BALB/c nude mice were used for xenograft experiments. OS-RC-2 cells were prepared at a concentration of 5 × 10⁶ cells/100 μL and injected subcutaneously into the mice. Tumor size was measured weekly. Four weeks post-injection, all animals were euthanized, and tumor volume and weight were recorded. The tumor volume was calculated as volume = (width^2^ × length × 0.5). Tumors were fixed in 10% formalin and embedded in paraffin for immunohistochemistry (IHC) analysis.

### Statistical analysis

Data are presented as mean ± standard deviation (SD) in bar plots, representing at least three independent experiments. An unpaired t-test was used to compare the mean differences between two independent samples. Two-way ANOVA analyzes the effects of two factors on the dependent variable. A *p*-value of <0.05 (two-tailed) was considered statistically significant (ns, *P* > 0.05; *P < 0.05; **P < 0.01; ***P < 0.001; ****P < 0.0001). Associations between MRPL12 K163 acetylation levels and clinicopathologic characteristics were evaluated using the chi-squared test. Univariate Cox regression and survival analyses were also performed. All statistical analyses were conducted using GraphPad Prism 8.0 (GraphPad Software, San Diego, CA) and IBM SPSS Statistics 25. Graph abstract was drawn by Figdraw.

## Supplementary information

Supplementary Figure 1

Supplemental Figure 1

western blot

## Data Availability

The datasets during and/or analysed during the current study available from the corresponding author on reasonable request.
