# Supplementary material for: MRPL12 K163 acetylation inhibits ccRCC via driving mitochondrial metabolic reprogramming
Source: Cell Death Dis. 2025 Aug 26;16(1):646. doi: 10.1038/s41419-025-07896-3 (PMC12381009; doi:10.1038/s41419-025-07896-3)
Supplement: Supplementary file 1 — Supplementary Figure 1 [file 41419_2025_7896_MOESM1_ESM.docx]

**Supplementary Figure 1**

EdU assays in OS-RC-2 and 786-O cells

(A) Effects of 2-DG and glucose on EdU analysis in OS-RC-2 and 786-O cells. Scale bar: 100 μm.

(B) Effects of SIRT5 knockdown and overexpression on EdU analysis. Scale bar: 100 μm.

(C) Effects of TIP60 knockdown and overexpression on EdU analysis. Scale bar: 100 μm. Significance levels: *P < 0.05; **P < 0.01; ***P < 0.001; ****P < 0.0001.
