# Supplementary figures and images for: MRPL12 K163 acetylation inhibits ccRCC via driving mitochondrial metabolic reprogramming
Source: Cell Death Dis. 2025 Aug 26;16(1):646. doi: 10.1038/s41419-025-07896-3 (PMC12381009; doi:10.1038/s41419-025-07896-3)

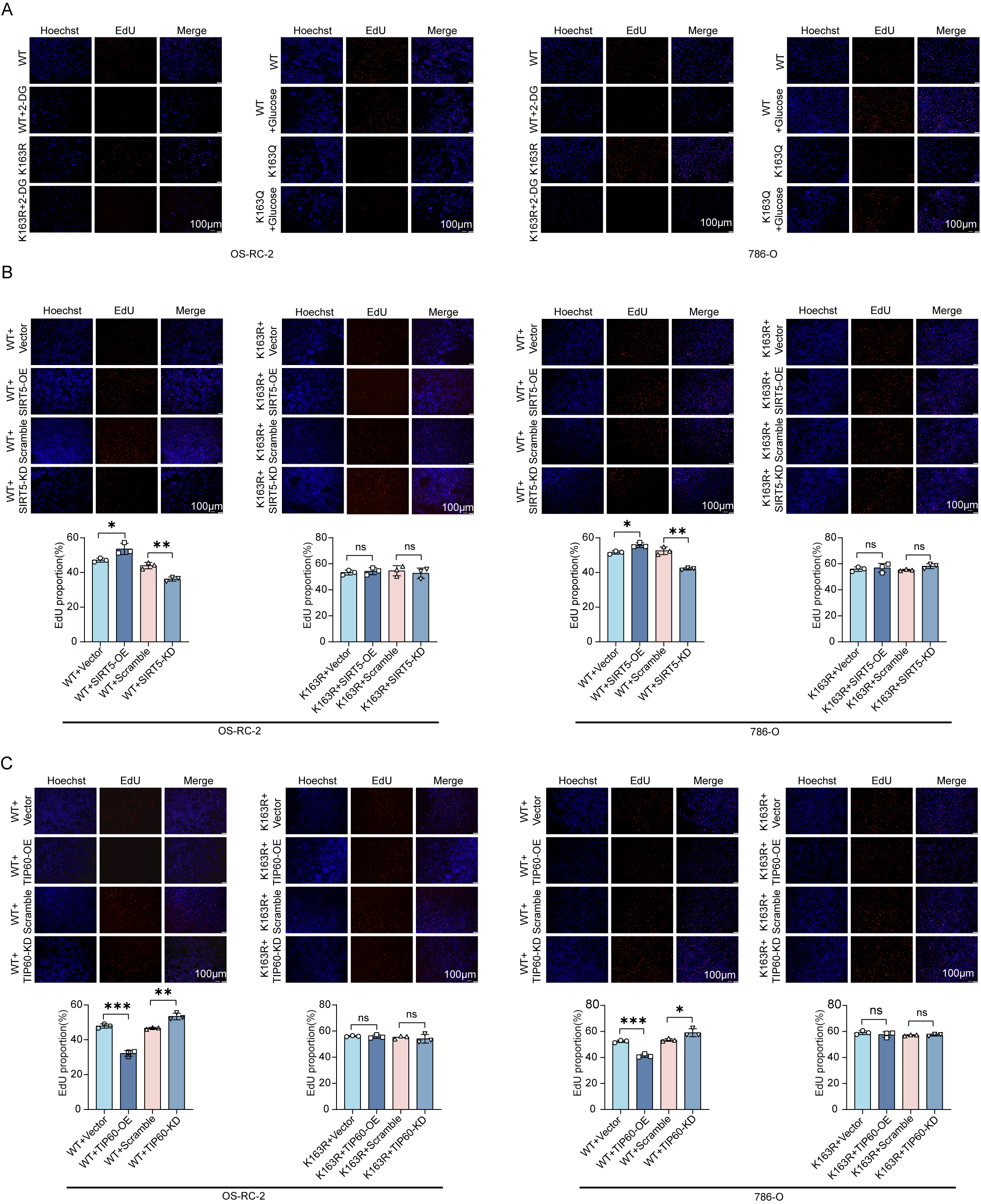

Supplement: Supplementary file 2 — Supplemental Figure 1 [file 41419_2025_7896_MOESM2_ESM.tif]
