# Supplementary material for: MRPL12 K163 acetylation inhibits ccRCC via driving mitochondrial metabolic reprogramming
Source: Cell Death Dis. 2025 Aug 26;16(1):646. doi: 10.1038/s41419-025-07896-3 (PMC12381009; doi:10.1038/s41419-025-07896-3)
Supplement: Supplementary file 3 — western blot [file 41419_2025_7896_MOESM3_ESM.pdf]

Figure 1

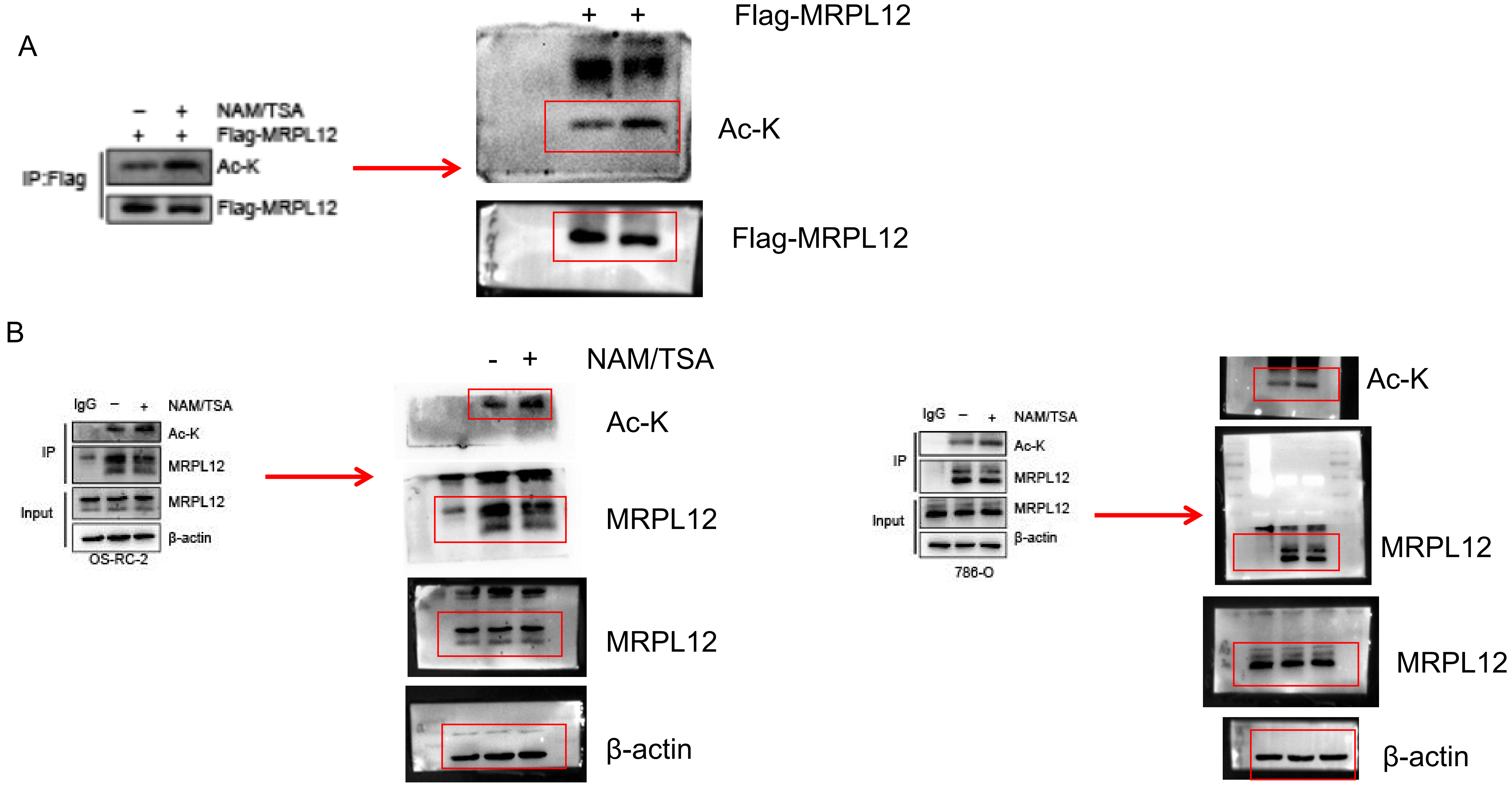
A
– + NAM/TSA
+ + Flag-MRPL12
IP:Flag
Ac-K
Flag-MRPL12
- + NAM/TSA
+ + Flag-MRPL12
Ac-K
Flag-MRPL12
B
IgG – + NAM/TSA
IP
Ac-K
MRPL12
Input
MRPL12
β-actin
OS-RC-2
- + NAM/TSA
Ac-K
MRPL12
MRPL12
β-actin
IgG – + NAM/TSA
IP
Ac-K
MRPL12
Input
MRPL12
β-actin
786-O
Ac-K
MRPL12
MRPL12
β-actin

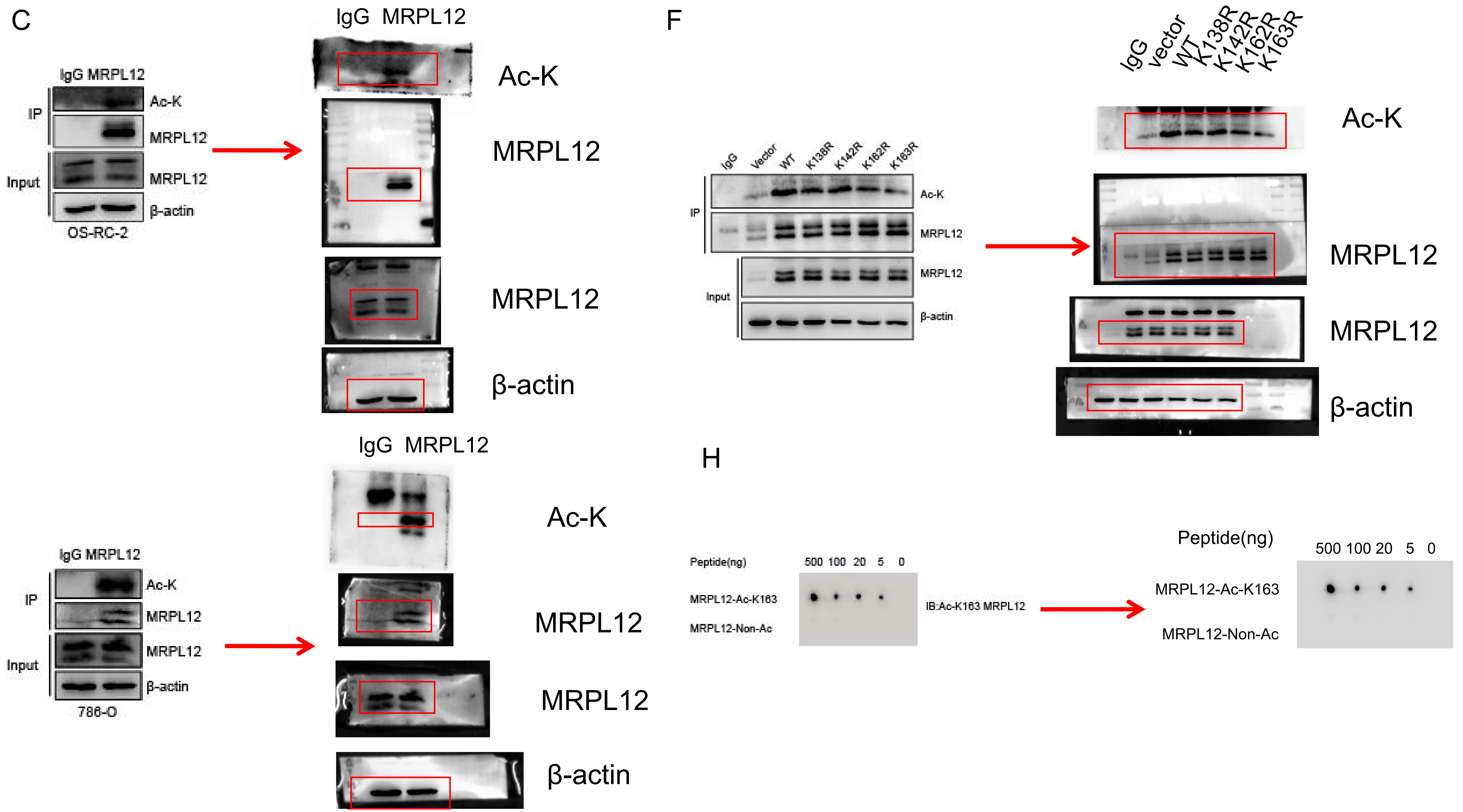
C
IgG MRPL12
IP
Ac-K
MRPL12
Input
MRPL12
β-actin
OS-RC-2
IgG MRPL12
Ac-K
MRPL12
MRPL12
β-actin
IgG MRPL12
IP
Ac-K
MRPL12
Input
MRPL12
β-actin
786-O
IgG MRPL12
Ac-K
MRPL12
MRPL12
β-actin
F
IgG
Vector
WT
K138R
K142R
K162R
K163R
IP
Ac-K
MRPL12
Input
MRPL12
β-actin
IgG
vector
WT
K138R
K142R
K162R
K163R
Ac-K
MRPL12
MRPL12
β-actin
H
Peptide(ng)
500 100 20 5 0
MRPL12-Ac-K163
MRPL12-Non-Ac
IB:Ac-K163 MRPL12
Peptide(ng)
500 100 20 5 0
MRPL12-Ac-K163
MRPL12-Non-Ac

Figure 2

H

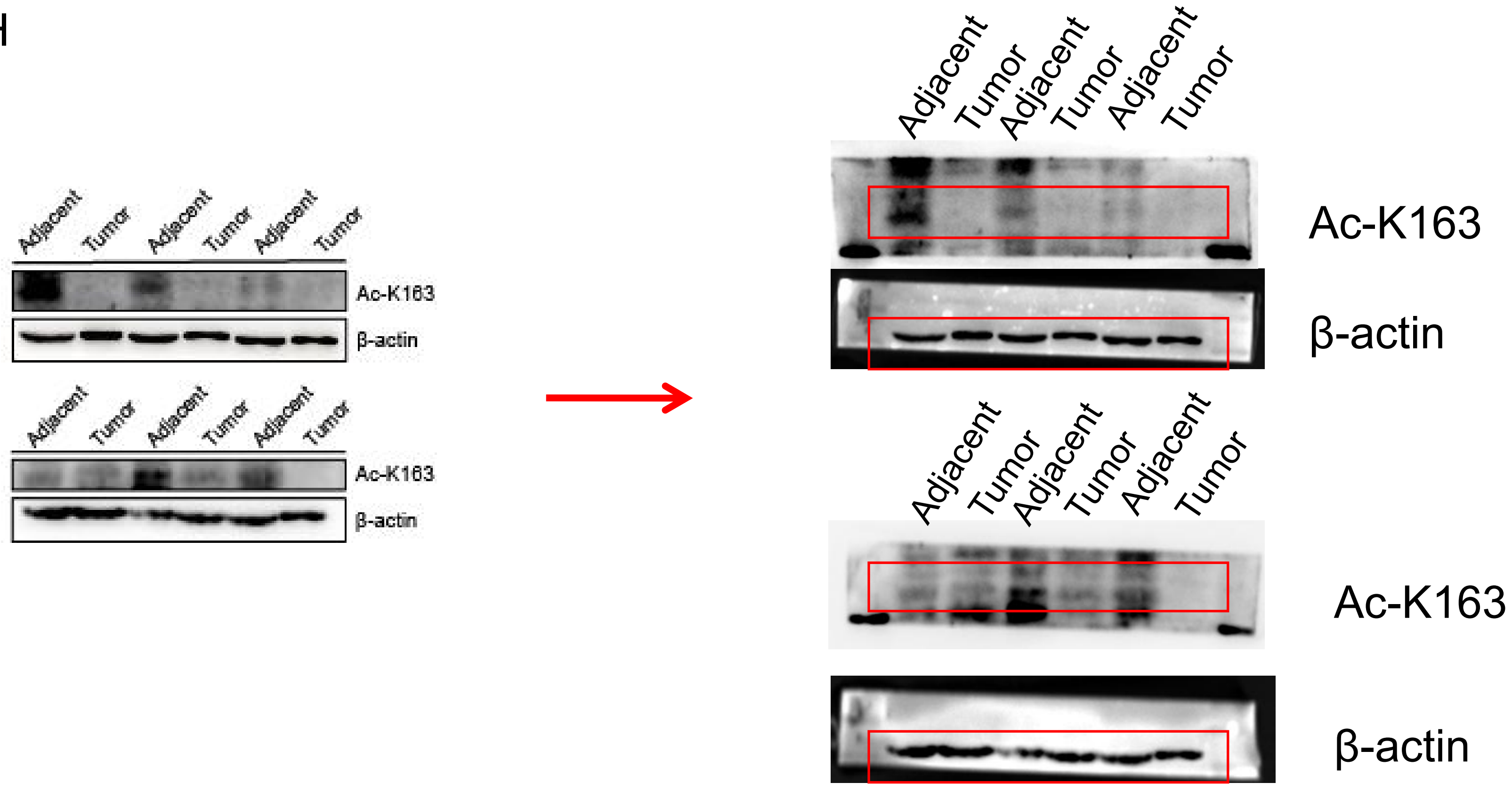
Adjacent
Tumor
Adjacent
Tumor
Adjacent
Tumor
Ac-K163
β-actin
Adjacent
Tumor
Adjacent
Tumor
Adjacent
Tumor
Ac-K163
β-actin
Adjacent
Tumor
Adjacent
Tumor
Adjacent
Tumor
Ac-K163
β-actin
Adjacent
Tumor
Adjacent
Tumor
Adjacent
Tumor
Ac-K163
β-actin

Figure 3

A

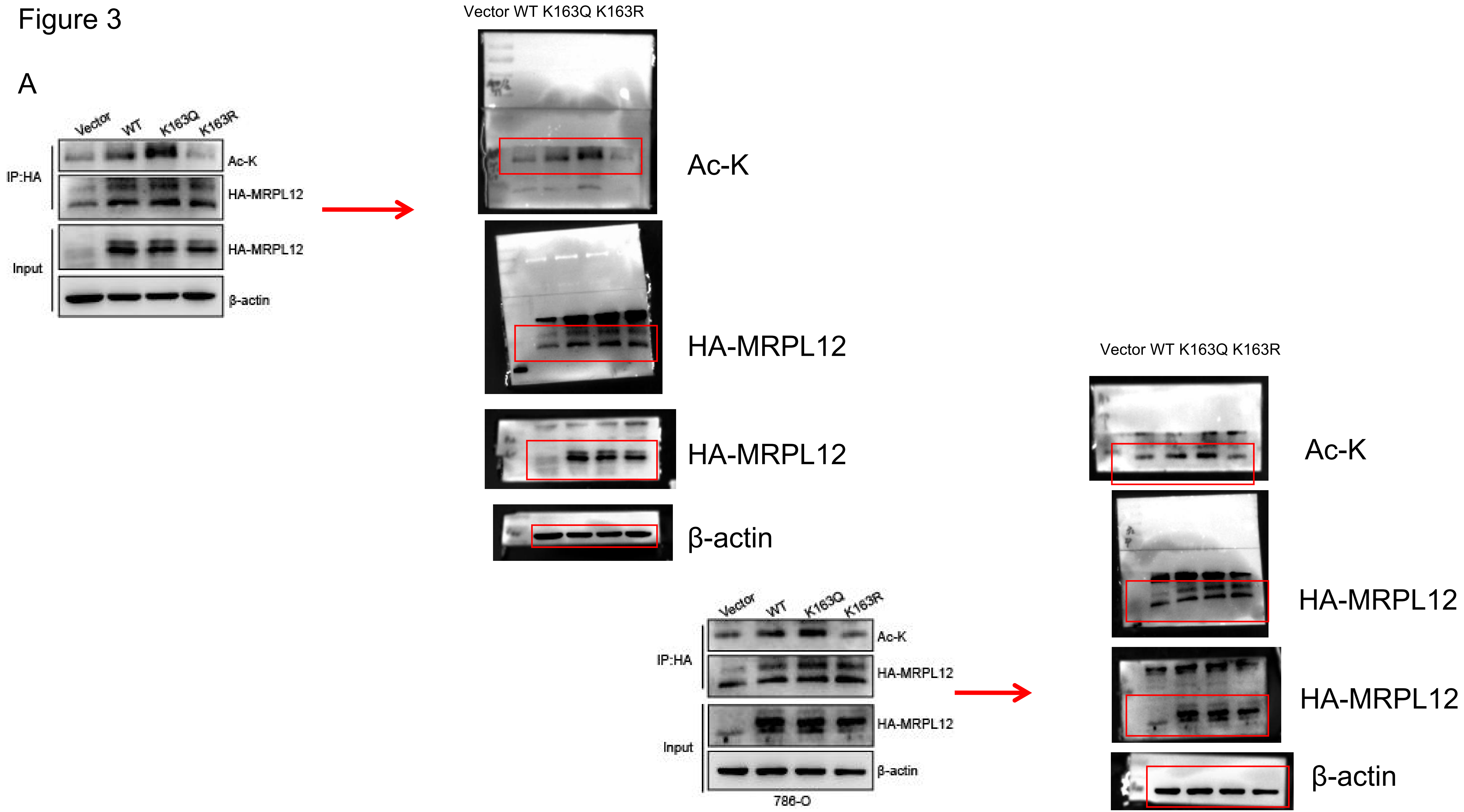

Vector WT K163Q K163R
IP:HA
Ac-K
HA-MRPL12
Input
HA-MRPL12
β-actin
Ac-K
HA-MRPL12
HA-MRPL12
β-actin
Vector WT K163Q K163R
IP:HA
Ac-K
HA-MRPL12
Input
HA-MRPL12
β-actin
786-O
Vector WT K163Q K163R
Ac-K
HA-MRPL12
HA-MRPL12
β-actin

Figure 3

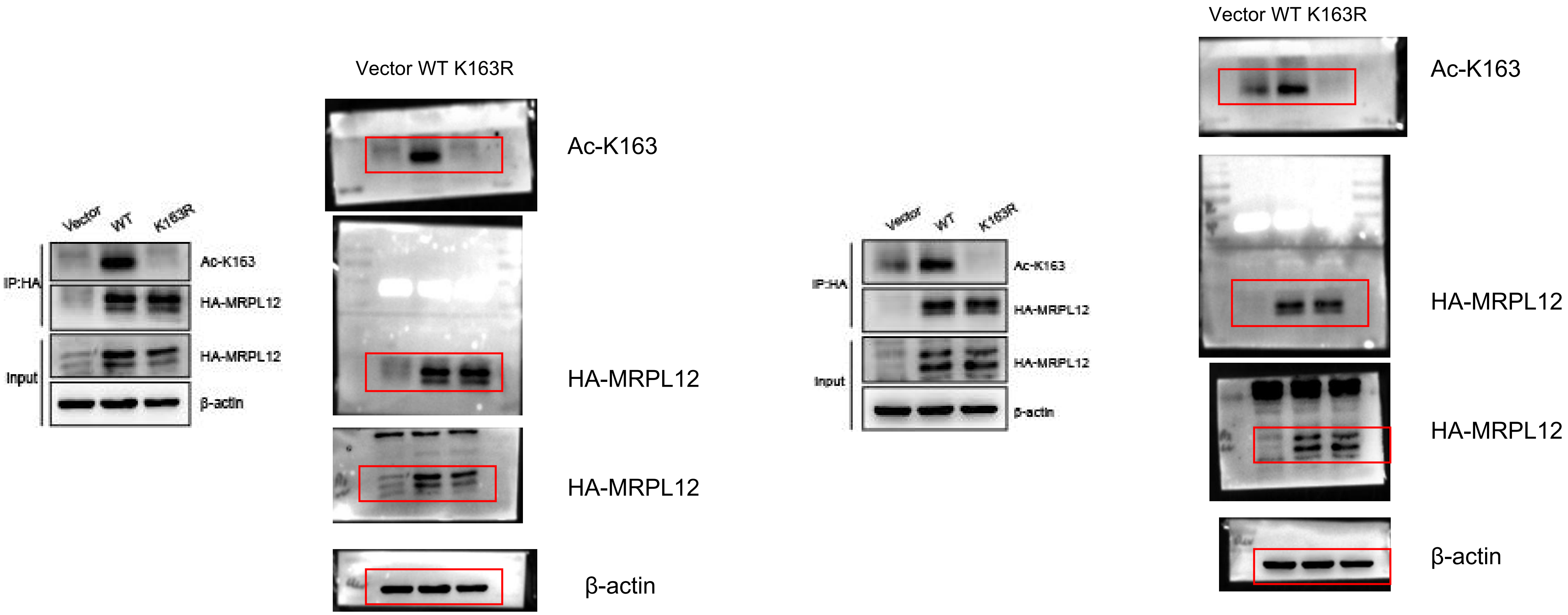
Vector
WT
K163R
IP:HA
Input
Ac-K163
HA-MRPL12
HA-MRPL12
β-actin
Vector WT K163R
Ac-K163
HA-MRPL12
HA-MRPL12
β-actin
Vector
WT
K163R
IP:HA
Input
Ac-K163
HA-MRPL12
HA-MRPL12
β-actin
Vector WT K163R
Ac-K163
HA-MRPL12
HA-MRPL12
β-actin

Figure 4

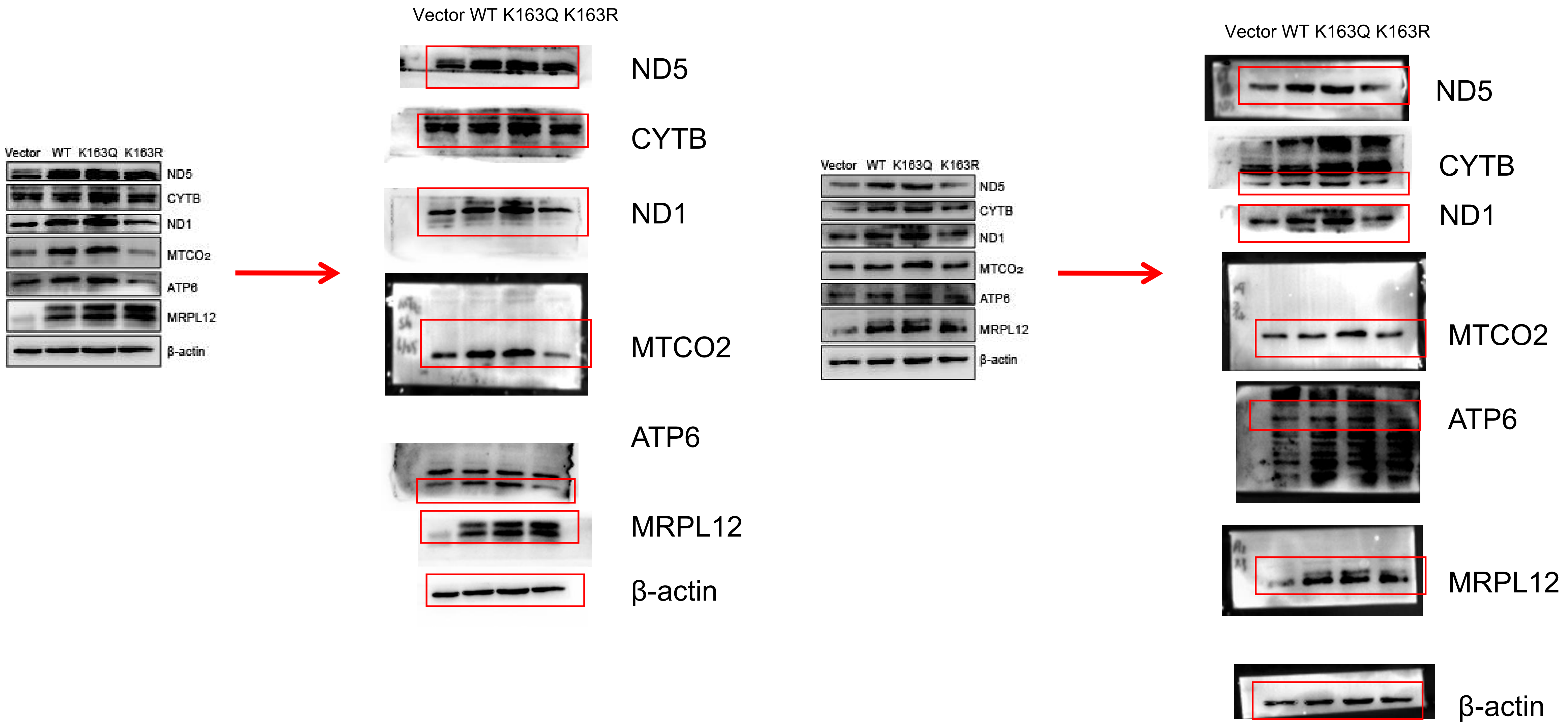
Vector WT K163Q K163R
ND5
CYTB
ND1
MTCO2
ATP6
MRPL12
β-actin
Vector WT K163Q K163R
ND5
CYTB
ND1
MTCO2
ATP6
MRPL12
β-actin
Vector WT K163Q K163R
ND5
CYTB
ND1
MTCO2
ATP6
MRPL12
β-actin
Vector WT K163Q K163R
ND5
CYTB
ND1
MTCO2
ATP6
MRPL12
β-actin

Figure 4

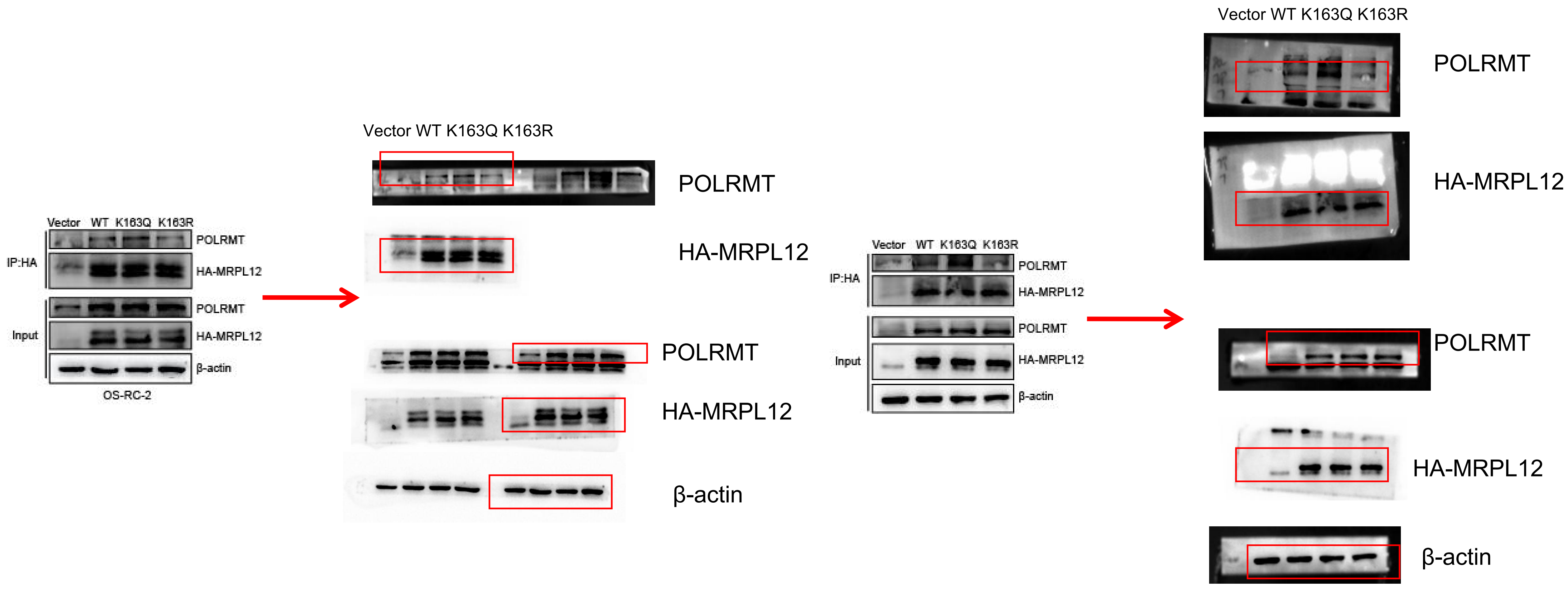

Vector WT K163Q K163R
IP:HA
POLRMT
HA-MRPL12
Input
POLRMT
HA-MRPL12
β-actin
OS-RC-2
Vector WT K163Q K163R
POLRMT
HA-MRPL12
POLRMT
HA-MRPL12
β-actin
Vector WT K163Q K163R
IP:HA
POLRMT
HA-MRPL12
Input
POLRMT
HA-MRPL12
β-actin
Vector WT K163Q K163R
POLRMT
HA-MRPL12
POLRMT
HA-MRPL12
β-actin

Figure 5

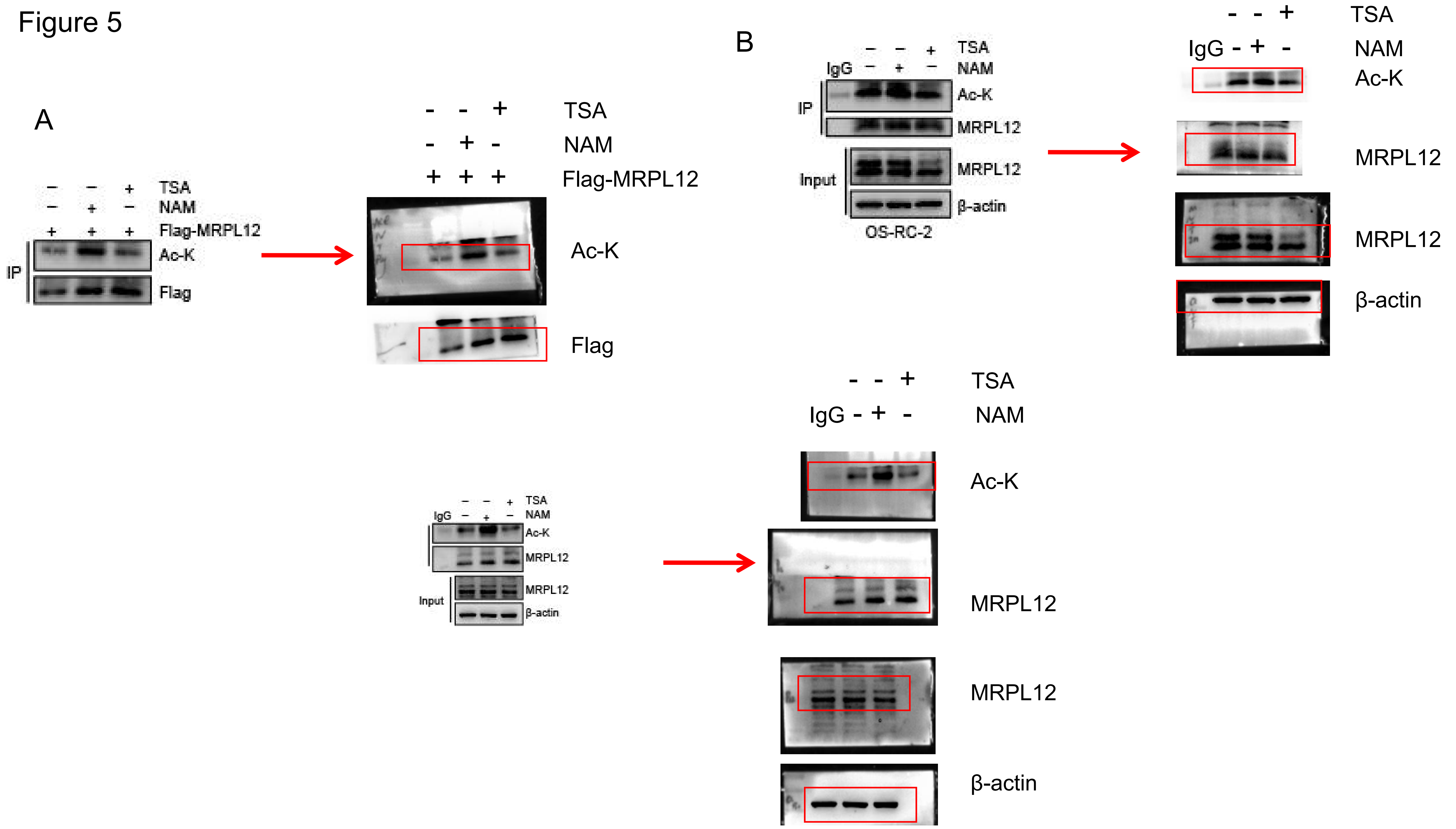

A
TSA
NAM
Flag-MRPL12
IP
Ac-K
Flag
B
IgG
OS-RC-2
Input
MRPL12
β-actin

Figure 5

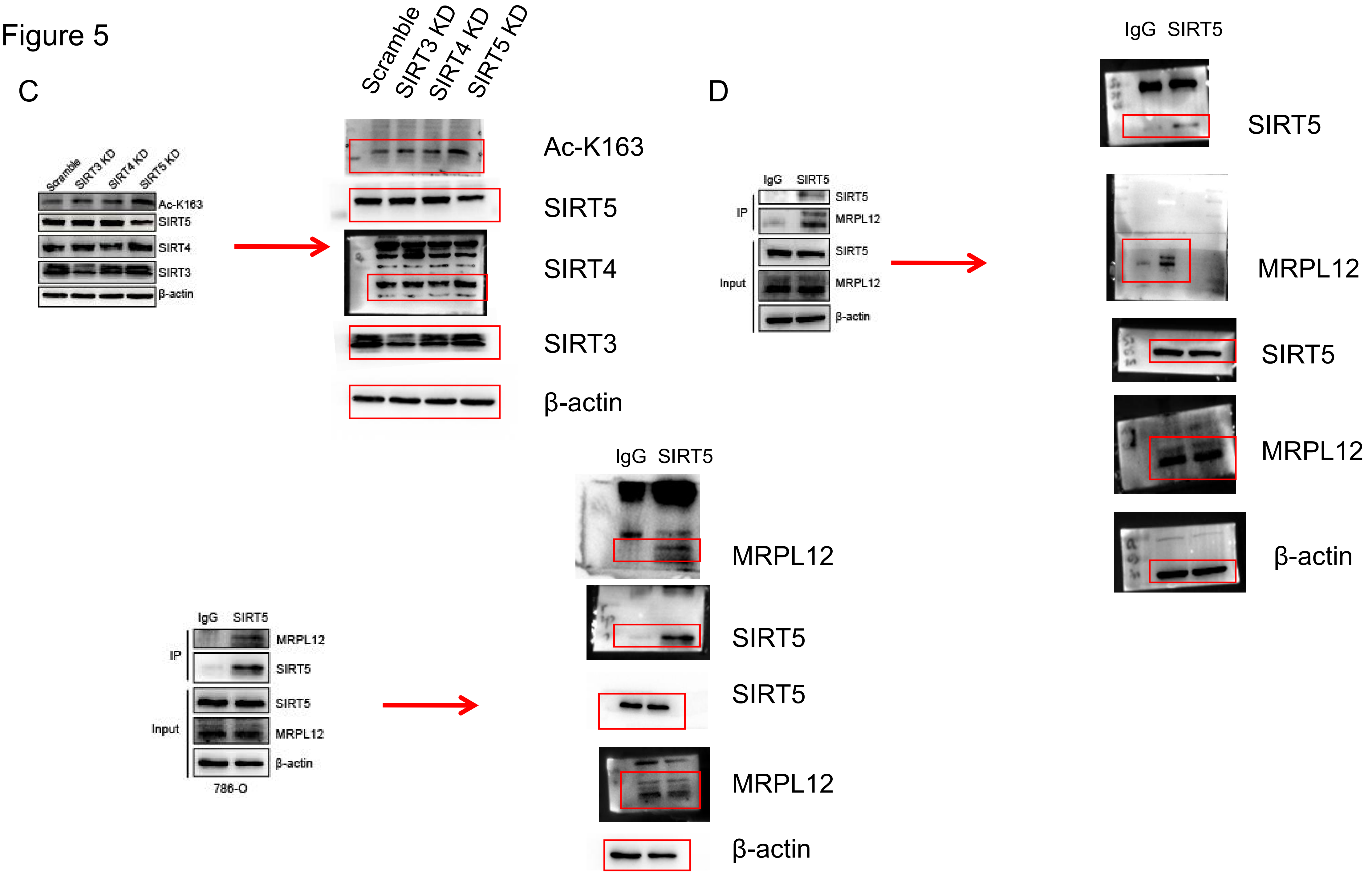
C
Scramble
SIRT3 KD
SIRT4 KD
SIRT5 KD
Ac-K163
SIRT5
SIRT4
SIRT3
β-actin
D
IgG
SIRT5
IP
Input
MRPL12
786-O

Figure 5

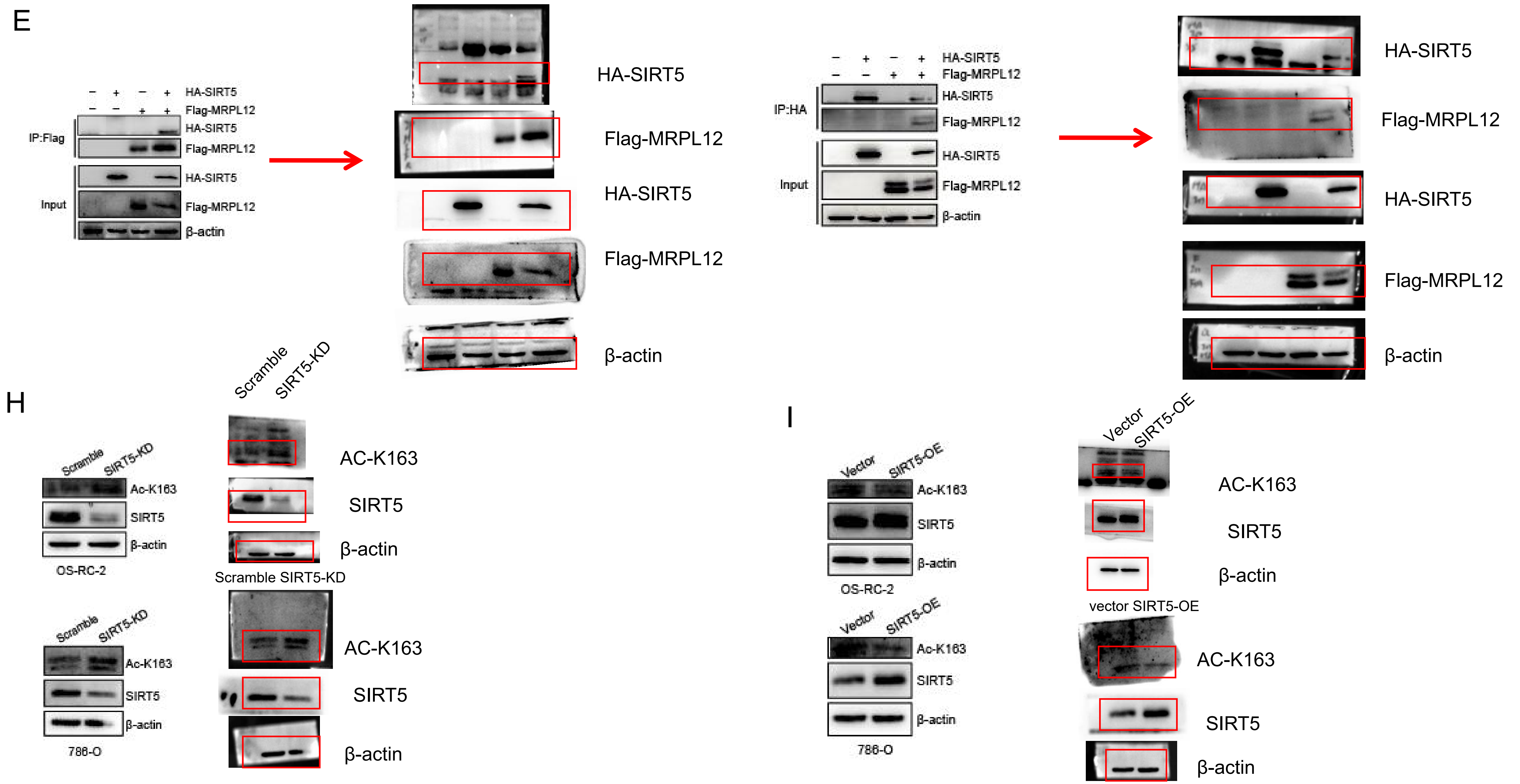
E
HA-SIRT5
Flag-MRPL12
IP:Flag
Input
β-actin
IP:HA
H
Scramble
SIRT5-KD
Ac-K163
AC-K163
SIRT5
OS-RC-2
786-O
Scramble SIRT5-KD
I
Vector
SIRT5-OE
vector SIRT5-OE

Figure 6

A

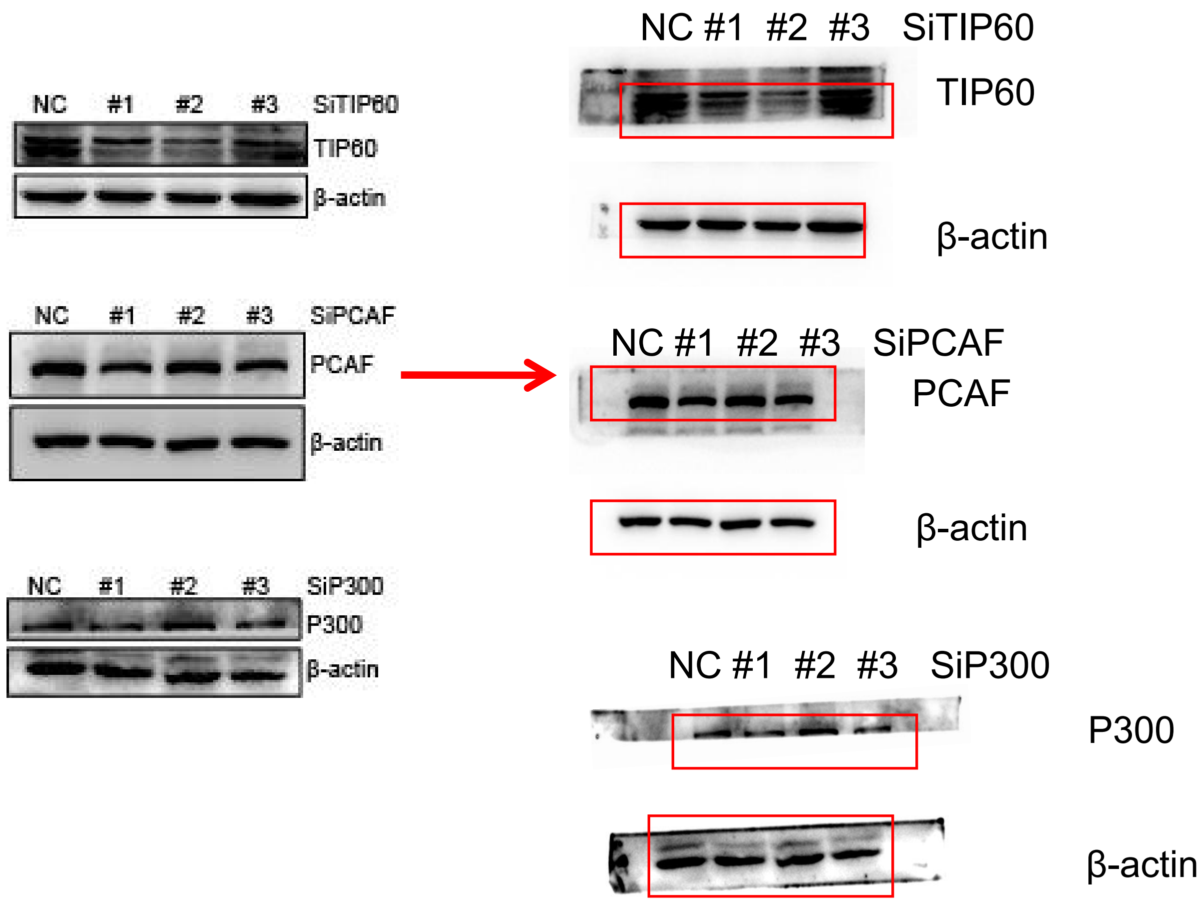
NC #1 #2 #3 SiTIP60
TIP60
β-actin
NC #1 #2 #3 SiPCAF
PCAF
β-actin
NC #1 #2 #3 SiP300
P300
β-actin
NC #1 #2 #3 SiTIP60
TIP60
β-actin
NC #1 #2 #3 SiPCAF
PCAF
β-actin
NC #1 #2 #3 SiP300
P300
β-actin

B

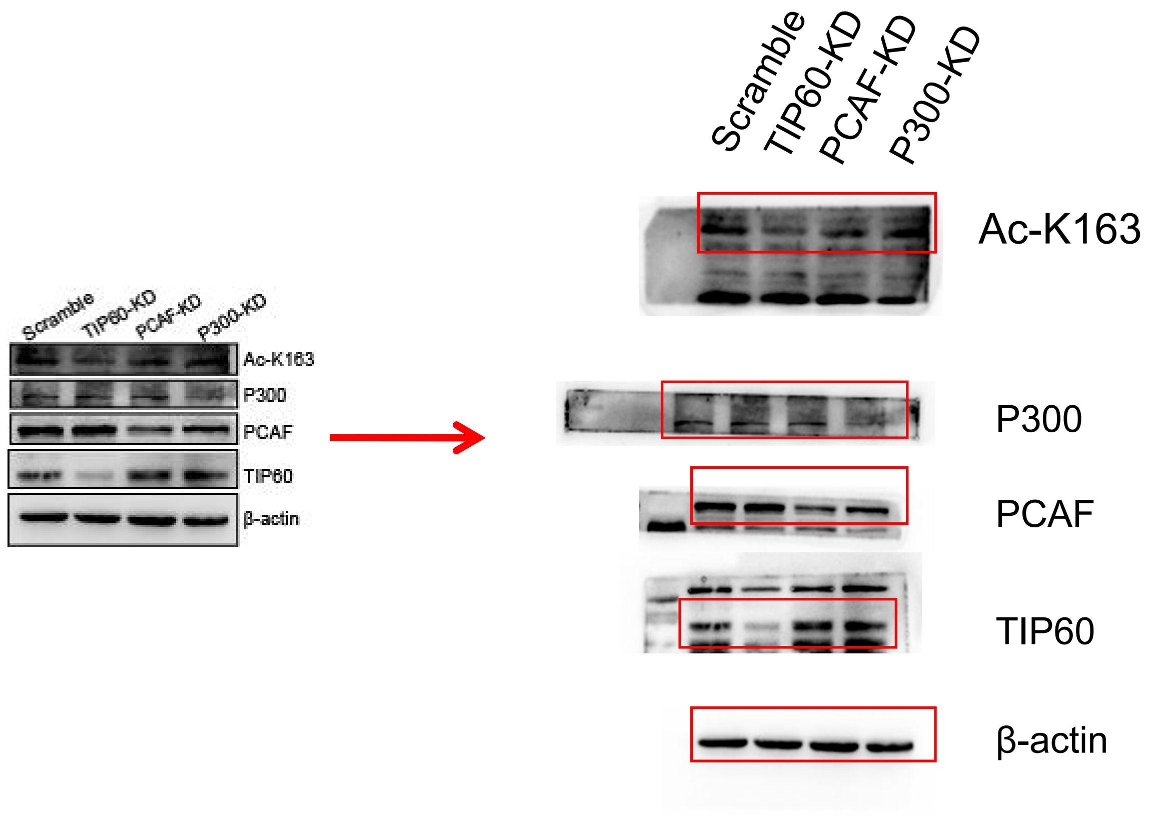
Scramble
TIP60-KD
PCAF-KD
P300-KD
Ac-K163
P300
PCAF
TIP60
β-actin
Scramble
TIP60-KD
PCAF-KD
P300-KD
Ac-K163
P300
PCAF
TIP60
β-actin

Figure 6

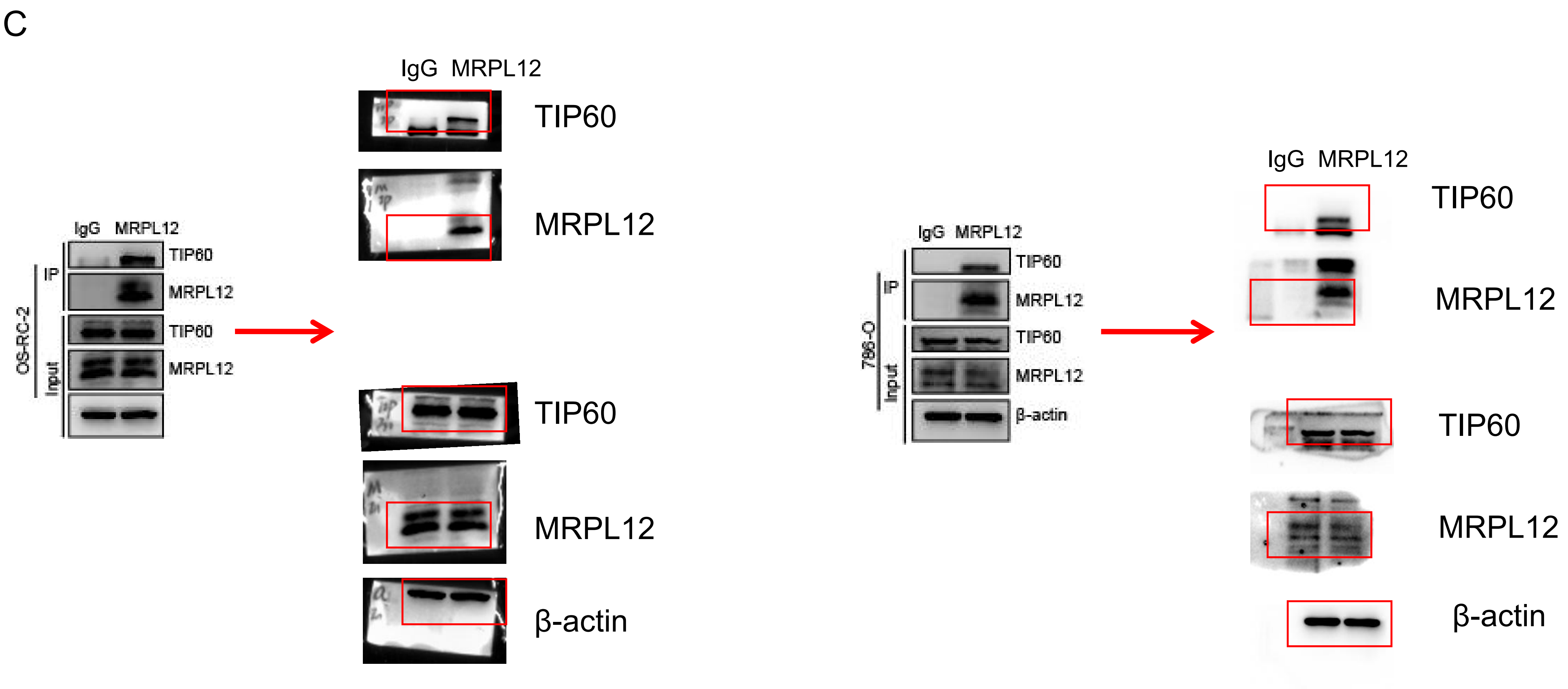
C
IgG MRPL12
TIP60
MRPL12
TIP60
MRPL12
OS-RC-2
IP
Input
TIP60
MRPL12
β-actin
IgG MRPL12
TIP60
MRPL12
TIP60
MRPL12
β-actin
786-O
IP
Input
β-actin

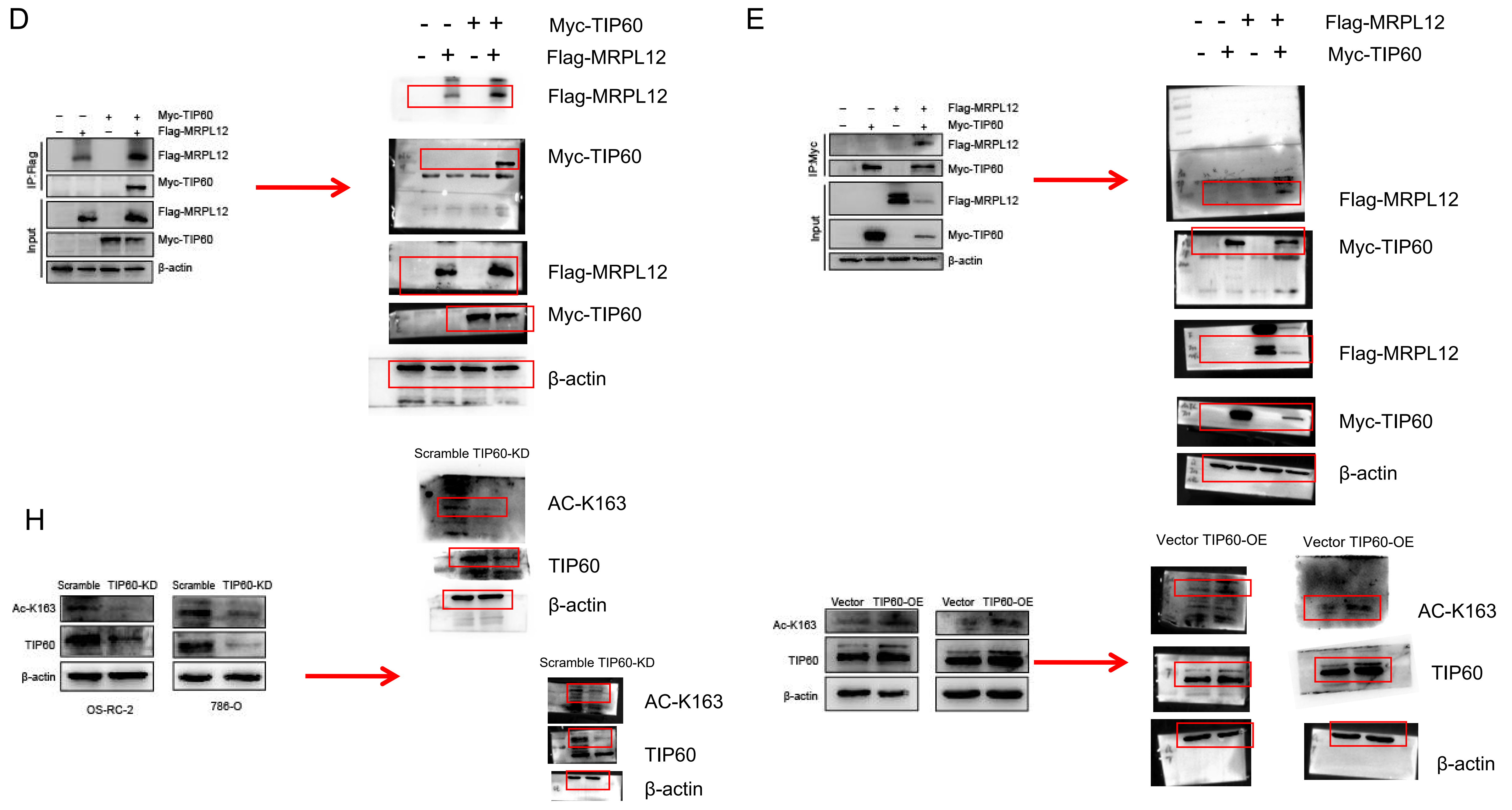
D
Myc-TIP60
Flag-MRPL12
IP:Flag
Input
Flag-MRPL12
Myc-TIP60
β-actin
E
IP:Myc
H
Scramble TIP60-KD
Ac-K163
AC-K163
TIP60
OS-RC-2
786-O
Vector TIP60-OE
